# ALKBH5 suppresses malignancy of hepatocellular carcinoma via m^6^A-guided epigenetic inhibition of LYPD1

**DOI:** 10.1186/s12943-020-01239-w

**Published:** 2020-08-10

**Authors:** Yunhao Chen, Yanchun Zhao, Junru Chen, Chuanhui Peng, Yanpeng Zhang, Rongliang Tong, Qiyang Cheng, Beng Yang, Xiaode Feng, Yuejie Lu, Haiyang Xie, Lin Zhou, Jian Wu, Shusen Zheng

**Affiliations:** 1 Division of Hepatobiliary and Pancreatic Surgery, Department of Surgery, First Affiliated Hospital, School of Medicine, Zhejiang University, Hangzhou, 310003 China; 2 NHC Key Laboratory of Combined Multi-organ Transplantation, Hangzhou, 310003 China; 3 Key Laboratory of the diagnosis and treatment of organ Transplantation, CAMS, Hangzhou, 310003 China; 4 Key Laboratory of Organ Transplantation, Hangzhou, 310003 Zhejiang Province China; 5 Hematology Department, the First Affiliated Hospital of Medical School of Zhejiang University, Hangzhou, 310003 China; 6 Zhejiang Provincial Research Center for Diagnosis and Treatment of Hepatobiliary Diseases (JBZX-202004), Hangzhou, 310003 China

**Keywords:** N6-methyladenosine (m^6^A), Hepatocellular carcinoma (HCC), ALKBH5, LYPD1

## Abstract

**Background:**

N6-methyladenosine (m^6^A) modification is an emerging layer of epigenetic regulation which is widely implicated in the tumorigenicity of hepatocellular carcinoma (HCC), offering a novel perspective for investigating molecular pathogenesis of this disease. The role of AlkB homolog 5 (ALKBH5), one of the m^6^A demethylases, has not been fully explored in HCC. Here we clarify the biological profile and potential mechanisms of ALKBH5 in HCC.

**Methods:**

Expression of ALKBH5 and its correlation with clinicopathological characteristics of HCC were evaluated using tissue microarrays and online datasets. And biological effects of ALKBH5 in HCC were determined in vitro and in vivo. Subsequently, methylated RNA immunoprecipitation sequencing (MeRIP-seq) combined with RNA sequencing (RNA-seq), and following m^6^A dot blot, MeRIP-qPCR, RIP-qPCR or dual luciferase reporter assays were employed to screen and validate the candidate targets of ALKBH5.

**Results:**

We demonstrated that ALKBH5 was down-regulated in HCC, and decreased ALKBH5 expression was an independent prognostic factor of worse survival in HCC patients. Functionally, ALKBH5 suppressed the proliferation and invasion capabilities of HCC cells in vitro and in vivo. Mechanistically, ALKBH5-mediated m^6^A demethylation led to a post-transcriptional inhibition of LY6/PLAUR Domain Containing 1 (LYPD1), which could be recognized and stabilized by the m^6^A effector IGF2BP1. In addition, we identified that LYPD1 induced oncogenic behaviors of tumors in contrast to ALKBH5. Dysregulation of ALKBH5/LYPD1 axis impelled the progression of HCC.

**Conclusion:**

Our study reveals that ALKBH5, characterized as a tumor suppressor, attenuates the expression of LYPD1 via an m^6^A-dependent manner in HCC cells. Our findings enrich the landscape of m^6^A-modulated tumor malignancy, and provide new insights into potential biomarkers and therapeutic targets of HCC treatment.

## Background

Hepatocellular carcinoma (HCC) is one of the most prevailing malignancies with poor long-term prognosis and high mortality [1]. Although diagnosis and treatment of HCC have considerably improved, the frequent recurrence or metastasis of HCC can hardly be prevented owing to the inadequate understanding of its sophisticated molecular pathogenesis [2, 3]. Therefore, it is quite essential to further explicate the biological mechanisms of HCC malignancy aiming to develop more effective therapeutic strategies.

Aberrations in epigenetic regulations such as DNA methylation, histone acetylation and RNA methylation, are crucial hallmarks of HCC carcinogenesis [4]. Emerging as the most common type of mRNA methylation in eukaryotes, N6-methyladenosine (m^6^A) modification attracted increasingly more attention nowadays [5]. The process of m^6^A methylation is reversible and dynamic regulated by methyltransferases (writers), demethylases (erasers) and effector proteins (readers) [6]. The canonical complex of writers called “WMM” is comprised of methyltransferase-like 3 (METTL3), methyltransferase-like 14 (METTL14) and Wilms tumor 1-associated protein (WTAP) [7], while identified erasers consist of fat-mass and obesity-associated protein (FTO) and AlkB homolog 5 (ALKBH5) [8, 9]. And readers are m^6^A-binding proteins including YT521-B homology (YTH) domain-containing family proteins (YTHDF1/2/3), YTH domain-containing proteins (YTHDC1/2), the insulin-like growth factor 2 mRNA-binding proteins family (IGF2BP1/2/3) and the heterogeneous nuclear ribonucleoprotein family (HNRNPs), which determine diverse comprehensive effects [10–12]. m^6^A modification accounts for far-ranging biological processes containing RNA metabolism, protein translation efficiency, transcription splicing, cell fate determination, immunologic homeostasis and tumorigenesis [13, 14].

Actually, it has been demonstrated that m^6^A modulation is extensively involved in the development of HCC [15]. For example, METTL14 is identified as a tumor suppressor via manipulating the m^6^A-mediated processing of pri-miR126 [16], while METTL3 enhances m^6^A-modification of SOCS2 to promote the evolution of HCC [17]. Besides, our previous work also emphasized the significance of WTAP in HCC through HuR-dependent post-transcriptional silencing of ETS1 [18]. And KIAA1429, a non-canonical writer, accelerates HCC pathogenesis via epigenetic regulation of GATA3 [19]. Moreover, Hou et al. substantiate that YTHDF2 suppresses tumor vasculature of HCC by facilitating the degradation of m^6^A-marked IL11 and SERPINE2 mRNA [20]. For m^6^A erasers, FTO has been reported to participate in HCC progression with controversial roles. Li et al. illustrate that FTO facilitates the tumorigenesis of HCC via modulating PKM2 demethylation [21]. However, a most recent study delineates that FTO, which is regulated by SIRT1-induced SUMOylation, functions as a tumor suppressor in HCC [22]. These outcomes underscore the complexity of m^6^A-mediated effects in HCC.

Nevertheless, few studies have investigated the role of another demethylase ALKBH5 in HCC tumorigenesis [23]. In our current study, we found that ALKBH5 was down-regulated in HCC, and lower ALKBH5 expression predicted poorer survival. Functionally, ALKBH5 inhibited the proliferation and invasiveness of HCC cells in vitro and in vivo. In addition, we verified that ALKBH5-modulated m^6^A modification, which is recognized by IGF2BP1, contributed to the post-transcriptional inactivation of LY6/PLAUR Domain Containing 1 (LYPD1). Furthermore, LYPD1 was subsequently identified as a novel oncoprotein in HCC. Thus ALKBH5-LYPD1 axis was closely involved in the malignancy of HCC. Our findings extend the understanding of m^6^A-driven machinery in HCC oncogenesis and highlight the significance of ALKBH5 in epitranscriptomic regulation.

## Materials and methods

### Patients and samples

Two HCC cohorts were included in this study, which was approved by Institutional Ethics Committee in First Affiliated Hospital of Zhejiang University. Cohort one contained 80 HCC patients who had undergone curative surgery from 2015 to 2018 in our hospital. Specimens of tumor and adjacent tissues were collected from these patients. RNA (70 pairs) or proteins (10 pairs) were isolated from frozen tissues for quantitative real-time PCR (qPCR) or western blotting assay to assess the expression of ALKBH5 in HCC. Cohort two consisted of 90 HCC patients which were the source of commercial tissue microarrays (TMA) supplied by Shanghai Outdo Biotech (LivH180Su07, Shanghai, China) together with integrated follow-up and clinical information data (illustrated in Table S1). This TMA cohort was employed to evaluate the role of ALKBH5 in HCC prognosis and construct the correlation of ALKBH5 and LYPD1 expression. Written informed consents were acquired from each patient relying on guidelines of the Declaration of Helsinki.

### Cell culture

The human HCC cell lines Huh7, MHCC97H, HCCLM3, HepG2, Hep3B, PLC/PRF/5, SMCC7721 and BEL7402 were obtained from the Shanghai Institutes of Biological Sciences (Shanghai, China). STR finger printing authentications of all employed HCC cells are available upon request. And here we present STR certificates for three of them (Huh7, MHCC97H and HCCLM3), which are mainly investigated in our study (Additional file 10). These cells were incubated at 37 °C in a 5% CO2 incubator (ThermoFisher, USA) with the humidified environment. And they were cultured with Minimum Essential Media (MEM, BI, Israel), which were routinely supplemented with 10% fetal bovine serum (FBS, BI), penicillin (100 units/ml) and streptomycin (100 μg/ml).

### m^6^A dot blot assay

Total RNA isolated from HCC cells or subcutaneous tumors was mixed in three times volume of incubation buffer and denatured at 65 °C for 5 min. Samples (400 ng, 200 ng or 100 ng) dissolved in SSC buffer (Sigma-Aldrich, Germany) were deposited on an Amersham Hybond-N+ membrane (GE Healthcare, USA) which was settled on the Bio-Dot Apparatus (Bio-Rad, USA). Then the membrane was crosslinked by UV light for 5 min, followed by the staining with 0.02% Methylene blue (Sangon Biotech, China). Scanning of blue dots were performed to show the input RNA content. And the membrane was hatched with m^6^A antibody (1:5000, Synaptic System, #202003) overnight at 4 °C. Dot blots were visualized by the imaging system after incubation with secondary antibody.

### Methylated RNA immunoprecipitation sequencing (MeRIP-seq) and data analysis

MeRIP-sequencing and following data analyses were mainly supported by Genesky Biotechnologies Inc. (Shanghai, China). HCCLM3 cells with stable overexpression of ALKBH5 and control cells transfected with an empty vector were collected (two replications; labeled as ALKBH5 and Vector, respectively). More than 250 μg of total RNA was extracted from each group, and mRNA was further purified with the NEBNext Poly(A) mRNA Magnetic Isolation Module (NEB#E7490) using oligo (dT) beads. Concentration and integrity of RNA were evaluated using NanoDrop Spectrophotometer and Agilent 2100 Bioanalyzer (Agilent, USA). The mRNA was then chemically fragmented into ~ 150 nts nucleotides with fragmentation buffer. After 10% of fragmented mRNA was saved as input, m^6^A-modified mRNA was immunoprecipitated with anti-m^6^A antibodies (Synaptic System, #202003) and eluted. RNA sequencing libraries for input mRNA (RNA-seq) and m^6^A-enriched mRNA (MeRIP-seq) were simultaneously constructed with the VAHTS Total RNA-seq (H/M/R) Library Prep Kit for Illumina (Box2&3, Vazyme#NR603), followed by the sequencing on Novaseq sequencer (Illumina, USA) with PE150 strategy. The MeRIP-seq data was analyzed based on the published standardized pipeline [24]. In brief, the raw data was aligned to human genome GRCh37/hg19 by the HISAT2 software (v2.0.5). Then m^6^A peaks were determined by the ExomePeak software (v2.6.0) and annotated according to the Ensembl database. Integrative Genomics Viewer (IGV) software was applied to present the visualization of the m^6^A peaks distribution. On the other hand, RNA-seq reads of input samples were normalized with Cufflinks (v2.2.1) [25], and Cuffdiff was employed to determine differentially expressed genes [26]. And major R codes during analyses were provided in Additional file 11.

### RNA immunoprecipitation (RIP)

RIP assay was conducted with Magna RIP Kit (Millipore, Germany) according to manufacturer’s illustrations. Briefly, magnetic beads were mixed with 5μg anti-ALKBH5 (Sigma-Aldrich, Germany) or IGF2BP1 (Abclonal, China) and anti-rabbit IgG (Millipore, Germany) before the addition of cell lysates (approximately 2*10^7^ cells for each sample). After the treatment of proteinase K, interested RNAs were eluted from immunoprecipitated complex and purified for further analysis using qPCR. Relative enrichment was normalized to the input: %Input =1/10 × 2^Ct [IP] – Ct [input]^.

### MeRIP-qPCR

MeRIP assay was performed with the Magna MeRIP™ m^6^A Kit (Millipore, Germany) to determine the m^6^A modification on individual transcripts. In brief, 150 μg total RNA was isolated from pretreated cells and randomly fragmented into a size of 100 or less nucleotides. RNA samples were then immunoprecipitated with magnetic beads pre-coated by 10 μg anti-m^6^A antibody (Millipore, Germany) or anti-mouse IgG (Millipore). And N6-methyladenosine 5′-monophosphate sodium salt (6.7 mM) were applied to elute the m^6^A-modified RNA fragments. Based on MeRIP-seq results, we focused on the sites of LYPD1 transcript where differential m^6^A peak was identified between ALKBH5-overexpressing cells and empty control cells (Fig. 5a). Specific primers were designed for MeRIP-qPCR analysis according to the information from MeRIP-seq and a motif-dependent m^6^A site predictor SRAMP (http://www.cuilab.cn/sramp) (Forward: AGCAGAATTGGCTGGTTTCG; reverse: AGCCCCAGTCTAAGTCCCA). Relative enrichment of m^6^A was normalized to the input: %Input =1/10 × 2^Ct [IP] – Ct [input]^.

### Statistical analysis

Statistical analysis was performed with the GraphPad Prism 8.0 (GraphPad, Inc., USA) and SPSS 22.0 (SPSS, Inc., USA) software. Experiments were independently repeated for at least three times. Representative data was exhibited as the means ± SD. Quantitative data was compared using two-tail Student t test, while qualitative data was evaluated by Chi-Square test. The overall and recurrence-free survival were analyzed with Kaplan–Meier method and log-rank test. And univariate and multivariate Cox regression models were employed to investigate independent prognostic factors. In addition, correlational analysis of gene expression was conducted with linear regression. *P*-values for every result were labeled on figures, and *P* < 0.05 was reckoned as statistically significant (*P < 0.05, **P < 0.01, ***P < 0.001, ****P < 0.0001).

More detailed methodology could be obtained in Supplementary Materials and Methods.

## Results

### Down-regulation of ALKBH5 is associated with poor prognosis of HCC

**Fig. 1:**
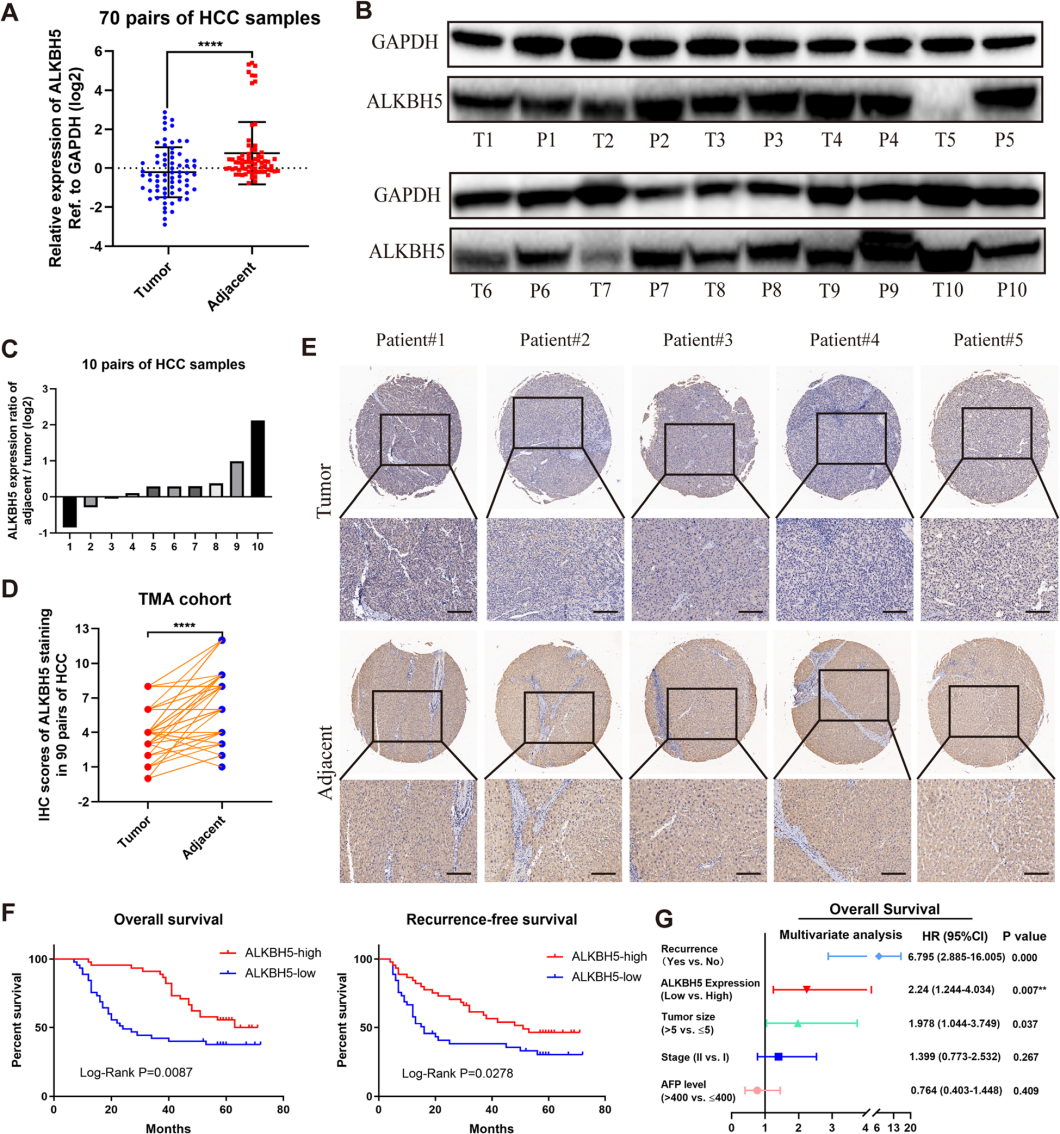
Down-regulated ALKBH5 expression correlates with poor outcomes of HCC patients. **a** The mRNA expression of ALKBH5 in tumor and normal tissues was measured based on 70 pairs of HCC samples (from cohort one); **b** Ten pairs of HCC samples (from cohort one) were subject to western blotting analysis of ALKBH5; **c** Grayscale analysis of ALKBH5 expression in **b** was conducted (calculated by log2 ratio of “adjacent/tumor pair”, normalized to GAPDH); **d** IHC scores of matched HCC and normal tissues (*n* = 90) were computed based on ALKBH5 staining (cohort two); **e** Representative images of ALKBH5 IHC staining in HCC samples were shown (scale bars, 100 μm; magnification, 100× and 200×); **f** Kaplan-Meier analysis of overall survival (left) and recurrence-free survival (right) of HCC patients based on ALKBH5 expression (*n* = 90). Cutoffs for grouping were determined by the median of IHC scores; **g** Multivariate analysis was employed for HCC patients using COX regression model based on those factors which were statistically significant in univariate analysis. Symbols and bars in forest plots correspond to HR and 95% CIs, respectively. T: tumor; P: para-tumor; HR: hazard rate; CI: confidence interval

To investigate the expression profile of ALKBH5 in HCC, we analyzed the mRNA and protein levels of ALKBH5 in HCC and matched adjacent tissues, and found that ALKBH5 was significantly down-regulated in HCC (Fig. 1a-c). And subsequent immunohistochemistry (IHC) staining with TMA from an independent HCC cohort confirmed these results (Fig. 1d, e). Besides, HCC patients with lower ALKBH5 expression obtained shorter overall survival (OS) and recurrence-free survival (RFS) (Fig. 1f). This finding was further validated by results from the Cancer Genome Atlas (TCGA) database (Additional file 5: Figure S1a). The expression of ALKBH5 also seemed to perform well in survival prediction of early-stage HCC patients (Additional file 5: Figure S1b). Moreover, loss of ALKBH5 was identified as an independent prognostic factor for HCC patients (HR = 2.24, *P* = 0.007) (Fig. 1g). It implies that dysregulation of ALKBH5 may be involved in the progression of HCC.

### ALKBH5 inhibits HCC proliferation in vitro and in vivo

**Fig. 2:**
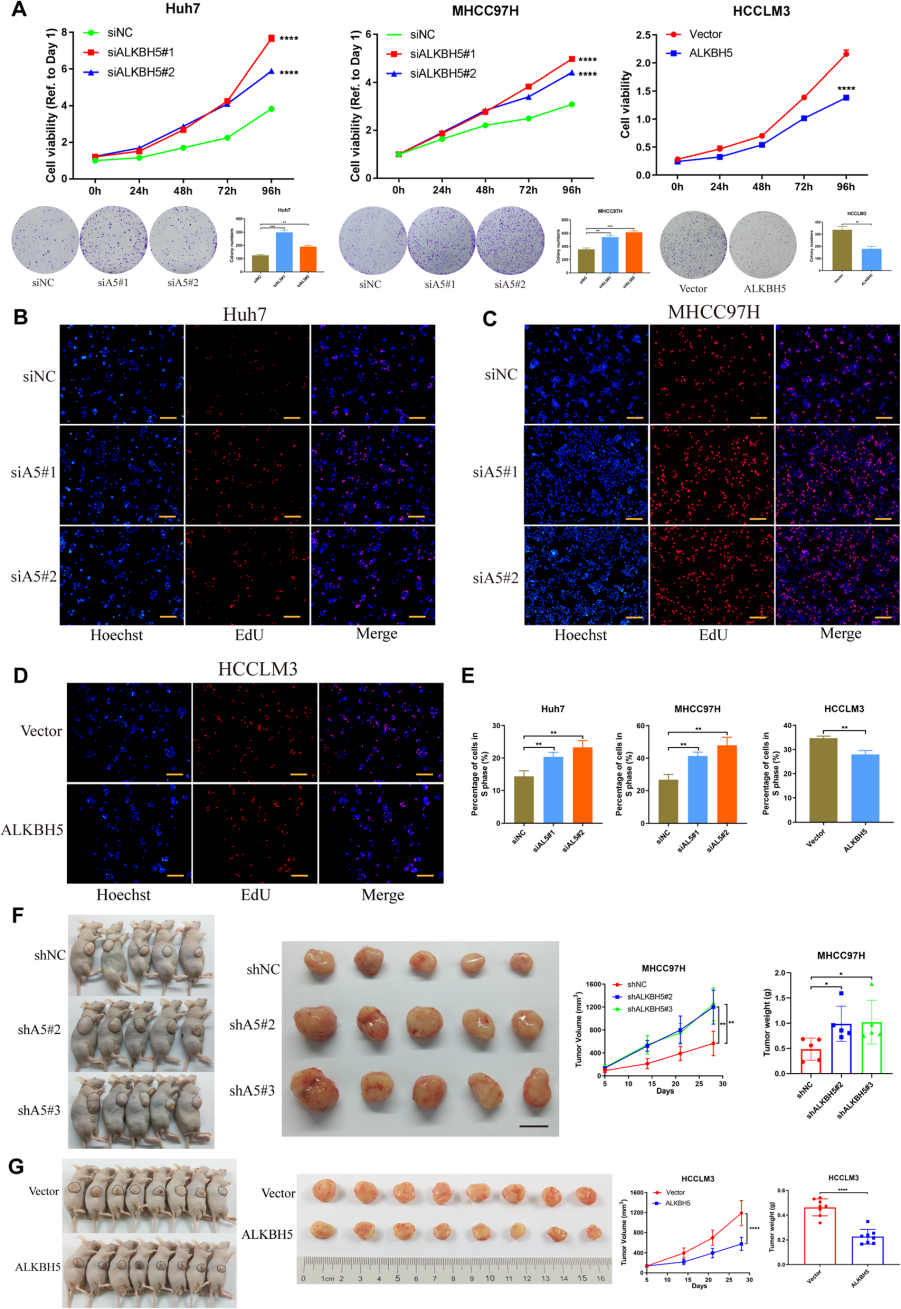
Inhibition of ALKBH5 drives HCC tumorigenesis. **a** CCK-8 and colony formation assays were applied to evaluate proliferation abilities of three HCC cell lines with knockdown or overexpression of ALKBH5. And histograms presented the colony numbers of each group; **b**, **c**, **d** and **e** EdU assays were conducted in three HCC cells to compare the percentage of cells in S phase (scale bars, 200 μm). Hoechst staining detected total cells, while EdU staining represented cells with active DNA replication. Representative images (**b-d**) and quantification data (**e**) were shown; **f** and **g** Tumor xenograft models were constructed with stable ALKBH5-knockdown (**f**, *n* = 5) or ALKBH5-overexpressing (**g**, *n* = 8) HCC cells and corresponding negative control cells (scale bar in **f**, 1 cm). Tumor sizes were recorded consecutively to establish tumor growth curves. Then tumors were collected from sacrificed mice and tumor weights were measured

To evaluate the functional roles of ALKBH5 in HCC, we firstly examined the expression of ALKBH5 in HCC cell lines (Additional file 5: Figure S1c, d). Huh7 or MHCC97H and HCCLM3 cells were chosen to establish ALKBH5-silencing and ALKBH5-overexpressing models, respectively. And the transfection efficiency was validated by qPCR and western blotting (Additional file 5: Figure S1e-h). As were indicated by CCK-8 and colony formation assays, knockdown of ALKBH5 enhanced the proliferation capability of HCC cells, while up-regulation of ALKBH5 showed the opposite effect (Fig. 2a). Similarly, EdU assay also demonstrated that ALKBH5 could suppress cell growth in vitro (Fig. 2b-e). Moreover, the re-expression of ALKBH5 was introduced into stable ALKBH5-knockdown cells (Additional file 6: Figure S2a, b). As expected, the restoration of ALKBH5 could rescue the enhanced proliferation capabilities induced by ALKBH5 loss in both Huh7 and MHCC97H cells (Additional file 6: Figure S2c, d), which was supported by EdU results as well (Additional file 6: Figure S2e-g).

To further address the anti-oncogenic role of ALKBH5 in HCC, we conducted in vivo experiments with subcutaneous tumor models. When ALKBH5 was silenced (Additional file 5: Figure S1i, j), volumes and weights of xenografted tumors increased compared with control group (Fig. 2f). On the contrary, ALKBH5 overexpression retarded tumor growth with considerably diminished tumor volumes and weights (Fig. 2g). And attenuation of PCNA, a marker of proliferation, was also detected along with the elevated expression of ALKBH5 (Additional file 6: Figure S2h). These results suggest that ALKBH5 exerts an inhibitory effect on HCC tumor growth in vitro and in vivo.

### ALKBH5 restrains migration/invasion abilities of HCC cells and inhibits metastasis in vivo

**Fig. 3:**
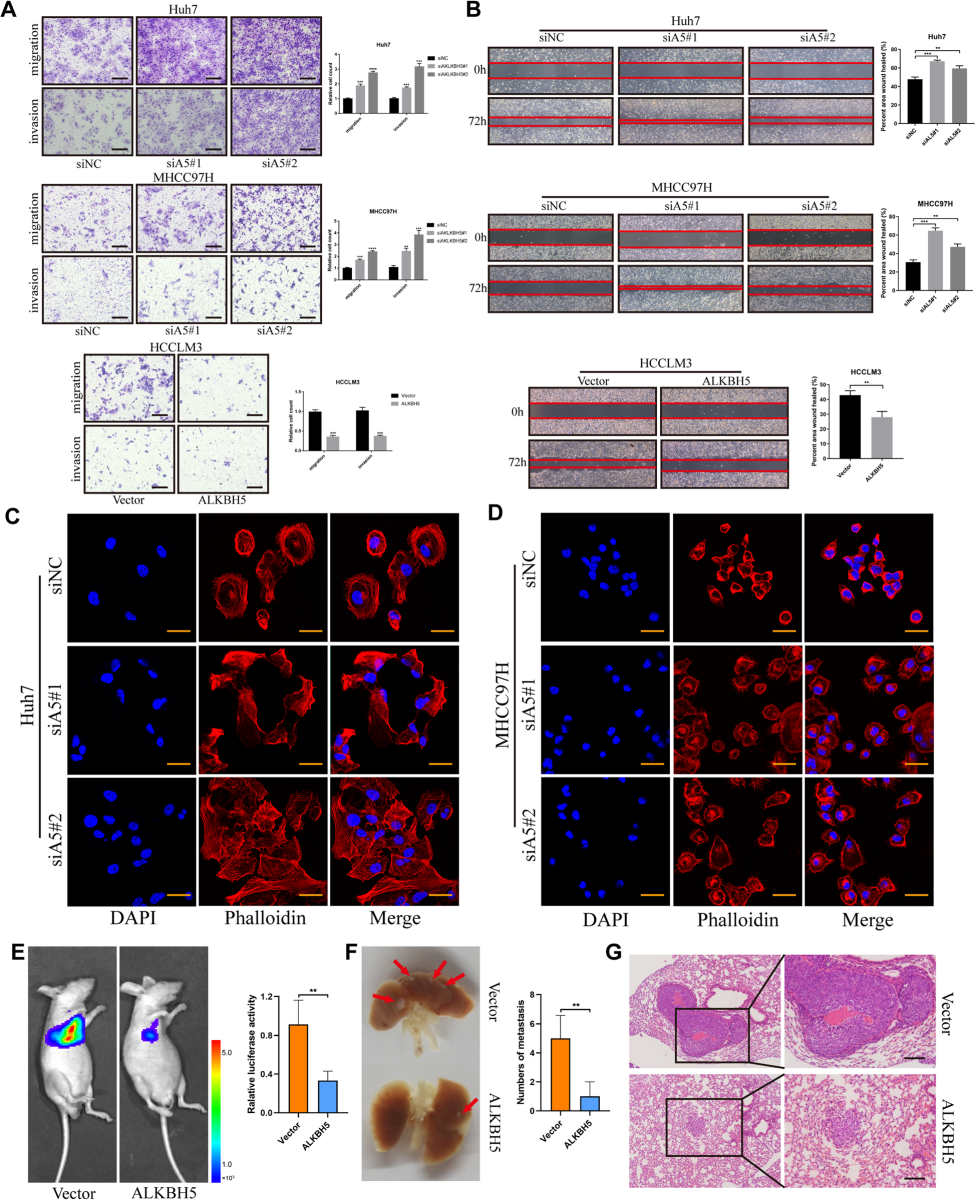
ALKBH5 abolishes migration/invasion capabilities of HCC cells in vitro and inhibits metastasis in vivo. **a** Transwell assays of Huh7, MHCC97H and HCCLM3 were applied to measure their migration and invasion abilities (scale bars, 200 μm). Bar charts showed the relative count (refer to negative control group) of cells which passed through the chamber membrane in each group (right); **b** Wound healing assays were conducted to compare the migration capabilities of three HCC cells after silencing or overexpression of ALKBH5. The difference in cell margin between 0 h and 72 h showed the moving track of cells; The percentage of healed area was quantified (right); **c** and **d** Alterations of cytoskeleton represented with immunofluorescent imaging were detected under the knockdown of ALKBH5 in Huh7 (**c**) and MHCC97H (**d**) cells. Phalloidin (red color) was applied for cytoskeleton staining, while DAPI (blue color) was used to mark the nuclei (scale bars, 30 μm). A divergent pattern of cytoskeleton with slenderer microtubules or microfilaments and more pseudopodia indicated a more flexible migrating style of cells; **e**, **f** and **g** HCCLM3 cells transfected with ALKBH5-overexpressing or control vector lentiviruses were injected into mice via tail vain to establish pulmonary metastasis models (*n* = 5). Representative in vivo images of mice were taken with quantification of luciferase activity in the lung region (**e**). Metastatic tumor foci in lungs were photographed and quantified (**f**), and their presence was further confirmed by HE staining (**g**) (scale bars, 100 μm)

We performed transwell assays and noticed that inhibition of ALKBH5 promoted both migration and invasion abilities of HCC cells, while overexpression of ALKBH5 impaired these phenotypes (Fig. 3a). Then the wound healing assay also indicated that ALKBH5 tended to attenuate migration of HCC cells (Fig. 3b). Interestingly, we always observed the altered cell morphology under microscope when ALKBH5 was silenced (data not shown). To verify whether this phenomenon was due to the reshaping of the cytoskeleton, phalloidin staining was performed subsequently. As expected, knockdown of ALKBH5 led to a looser and more divergent pattern of cytoskeleton through the rearrangement of microtubules and microfilaments (Fig. 3c, d), which denoted a more active migrating form.

To clarify the effects of ALKBH5 on HCC metastasis in vivo, ALKBH5-overexpressing and negative control HCCLM3-luc cells were implanted into BALB/c mice via tail vein injection, followed by the bioluminescence imaging. It seemed that activation of ALKBH5 damaged the metastatic potential of HCC cells with lower luciferase activity and less pulmonary metastasis (Fig. 3e, f), which was confirmed by HE staining results (Fig. 3g). In contrast, silencing of ALKBH5 promoted the metastasis of HCC (Additional file 6: Figure S2i). Therefore, ALKBH5 suppresses the migration/invasion abilities of HCC cells in vitro and their metastatic capabilities in vivo.

### MeRIP-seq combined with RNA-seq reveals LYPD1 as a target of ALKBH5

**Fig. 4:**
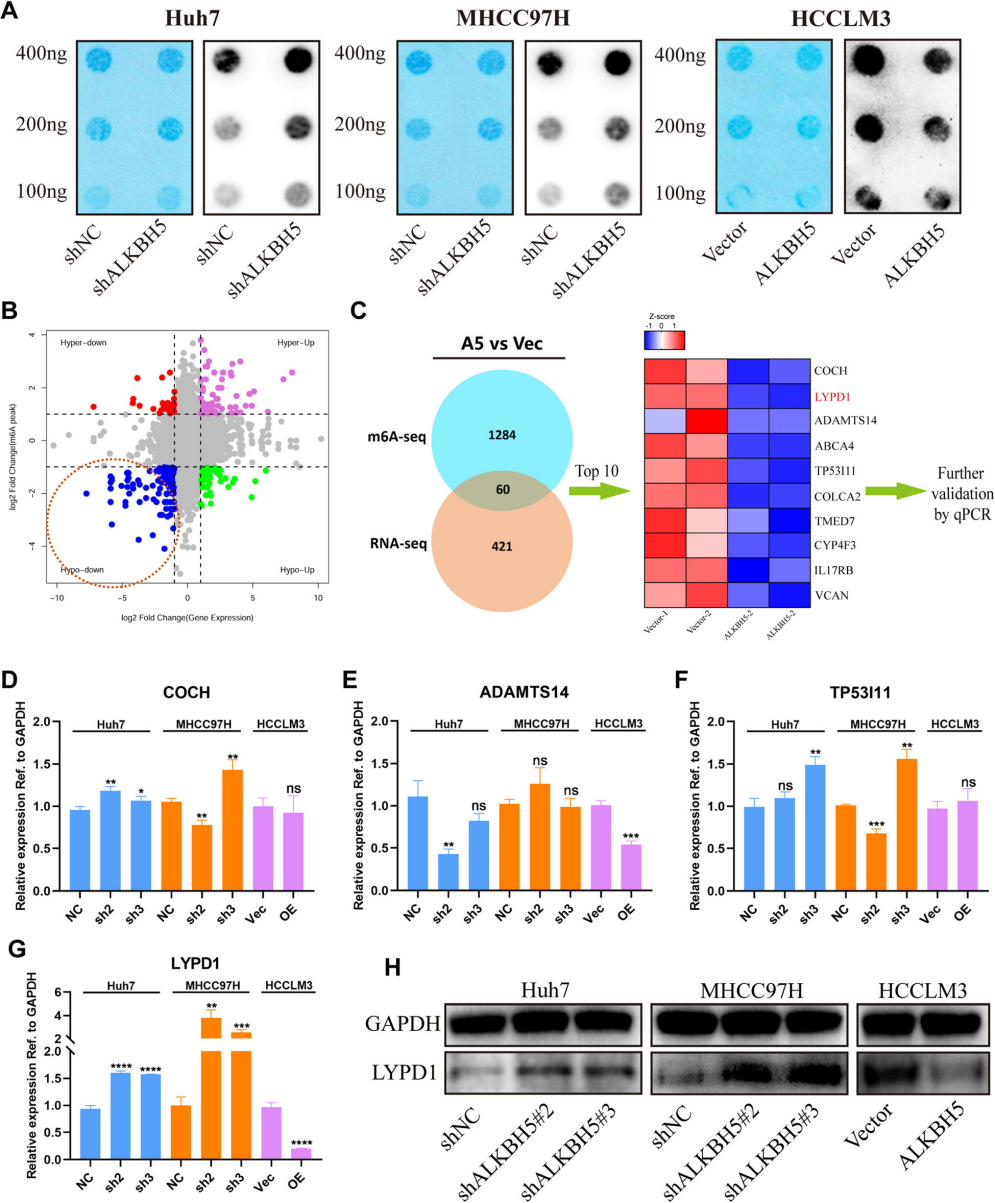
LYPD1 is identified as the candidate target of ALKBH5. **a** Global m^6^A level of RNA extracted from ALKBH5-knockdown or -overexpressing HCC cells was measured via m^6^A dot blot assays. RNAs were serially diluted and loaded equally with the amount of 400 ng, 200 ng and 100 ng. And methylene blue staining (left) was used to detect input RNA, while the intensity of dot immunobloting (right) represented the level of m^6^A modification. **b** The starplot showed the distribution of genes with both differential (hyper or hypo) m^6^A peaks (Y axis; fold change > 1.5 or < 2/3, *P* < 0.05) and differential (up or down) expression (X axis; fold change > 2 or < 0.5, *P* < 0.05) in ALKBH5-overexpressing group compared with control group. The blue dots highlighted by a circle represented down-regulated transcripts with the reduced abundance of m^6^A upon overexpression of ALKBH5, which were selected for the following investigations. **c** A schematic diagram showed the screening criterion for ALKBH5 targets. Results of MeRIP-seq (blue circle) and RNA-seq (brown circle) were combined using the Venn diagram. The overlap contained 60 transcripts which were influenced by ALKBH5 in both m^6^A content and expression. And the prescreening was based on expression level. The top 10 differentially expressed genes showed in the heat map (red indicated up-regulation and blue indicated down-regulation) were subject to following validation using qPCR. **d**, **e**, **f** and **g** RNA level of COCH (**d**), ADAMTS14 (**e**), TP53I11 (**f**) and LYPD1 (**g**) were examined in ALKBH5-silenced or -overexpressing cells, respectively. Those genes which were consistently validated in all three HCC cell lines were subject to further studies; **h** Protein level of LYPD1 was measured in ALKBH5-silenced Huh7 and MHCC97H cells or ALKBH5-overexpressing HCCLM3 cells

We firstly applied dot blot assays to examine the role of ALKBH5 in modulating m^6^A modification. Loss of ALKBH5 led to a convincingly increased m^6^A level in both Huh7 and MHCC97H cells, while ALKBH5 overexpression generated the opposite result (Fig. 4a). And the same conclusion could be obtained from the analysis of subcutaneous tumors (Additional file 6: Figure S2j).

To find out the precise mechanisms underpinning the observed ALKBH5-dependent phenotypes, an integrated approach combining MeRIP-seq and RNA-seq was employed using stable ALKBH5-overexpressing and vector-transfected HCCLM3 cells. MeRIP-seq revealed 1538 differential m^6^A peaks with reduced abundance (1344 corresponding transcripts) when ALKBH5 was up-regulated. Meanwhile, RNA-seq uncovered 481 down-regulated transcripts upon ALKBH5 overexpression.

We attached more importance to oncogenes whose methylation patterns and expression levels were regulated by ALKBH5. Therefore, merely those transcripts owning both hypo-m^6^A-peaks and decreased expression upon ALKBH5 overexpression were selected for following investigations (Fig. 4b). To further narrow down the scope of candidates, we focused on the top 10 genes from the overlap, namely COCH, LYPD1, ADAMTS14, ABCA4, TP53I11, COLCA2, TMED7, CYP4F3, IL17RB and VCAN, listed in ascending order of expression fold change. They were subject to preliminary validation in ALKBH5-silencing or -overexpressing cells by qPCR (Fig. 4c). Intriguingly, only LYPD1 was consistently found to be inversely regulated by ALKBH5 in all three HCC cells (Fig. 4d-g; Additional file 7: Figure S3a-e), which was further confirmed by western blotting results (Fig. 4h). Taken together, LYPD1 may be the direct downstream target of ALKBH5.

### ALKBH5-regulated m^6^A modification abolishes stability of LYPD1 via an IGF2BP1-dependent manner

**Fig. 5:**
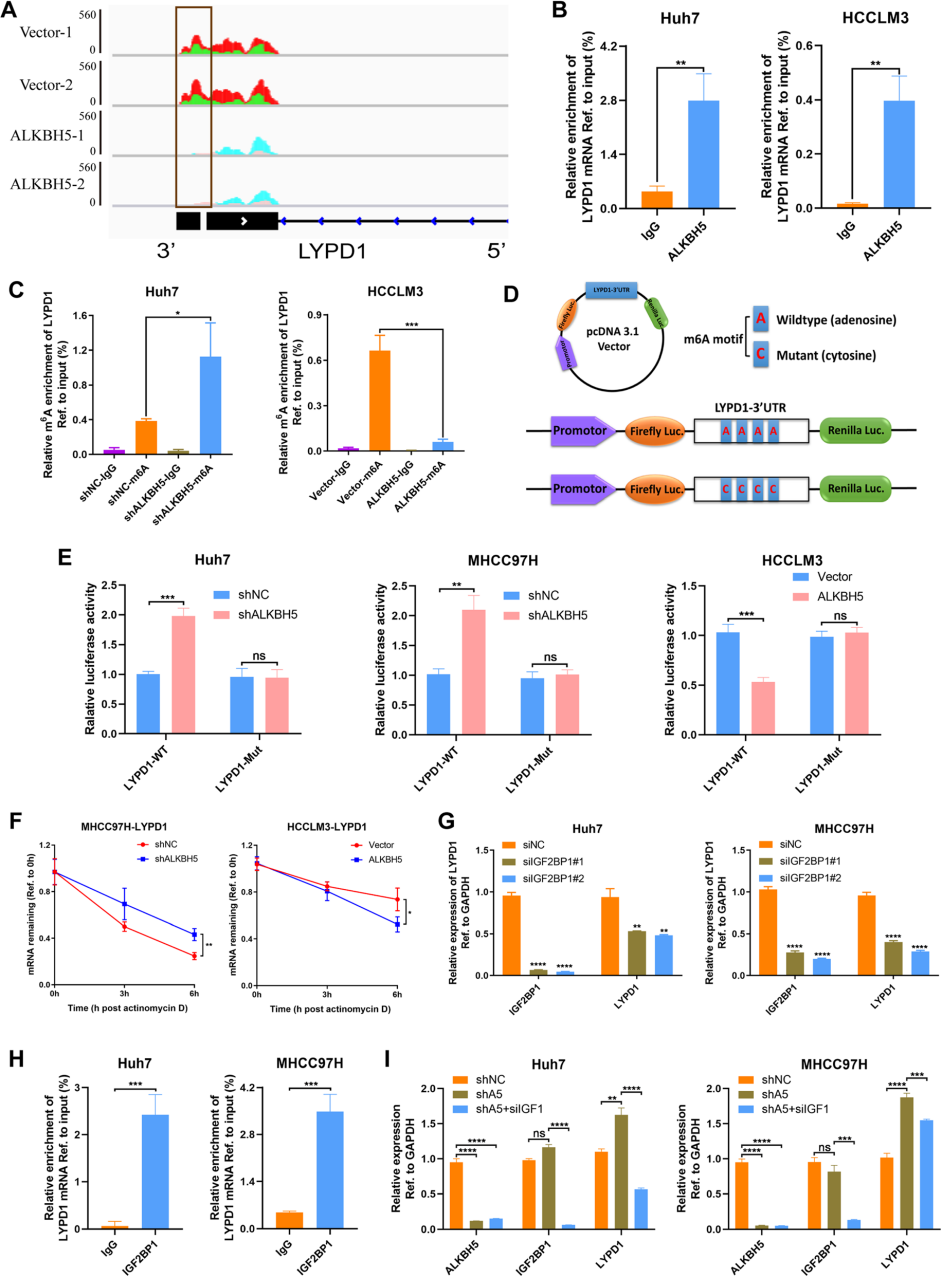
ALKBH5 impairs the stability of LYPD1 mRNA via an IGF2BP1-m^6^A-dependent pattern. **a** m^6^A abundance on LYPD1 mRNA in negative control or ALKBH5-overexpressing HCCLM3 cells was plotted by the IGV. Green and pink colors show the m^6^A signals of input samples, while red and blue stand for signals of IP samples. The range of signals in all groups was normalized to a 0–560 scale. At the same position, m^6^A peaks of IP group over input group were recognized as the genuine m^6^A level. Black blocks below figure indicated the sites where the m^6^A level differed between two groups, and the most remarkable location was highlighted with a gray pane. **b** Relative enrichment of LYPD1 mRNA associated with ALKBH5 protein was identified by RIP assays using anti-IgG and anti-ALKBH5 antibodies. The IgG group was a negative control to preclude nonspecific binding. The Y axis represented the percent of input for each IP sample according to the formula: %Input =1/10*2^Ct [IP] – Ct [input]^. **c** m^6^A modification of LYPD1 was detected by MeRIP-qPCR analysis using anti-IgG and anti-m^6^A antibodies. Relative m^6^A enrichment of LYPD1 mRNA for each IP group was normalized to input. Silencing of ALKBH5 induced an increase m^6^A abundance on LYPD1 compared with control group, while ALKBH5 overexpression led to the opposite result; **d** Graphical explanation for construction of luciferase reporters. The wild-type (full-length) or mutant (m^6^A motif mutated) sequence of LYPD1–3’UTR was inserted into a pcDNA3.1 vector between Firefly and Renilla elements. Relative luciferase activity was computed by the ratio of Firefly and Renilla luciferase values. **e** Relative luciferase activity of Huh7, MHCC97H and HCCLM3 cells transfected with the LYPD1-wild type or -mutated construct was measured, with normal or altered expression of ALKBH5; **f** ALKBH5-silenced or -overexpressed cells were treated with actinomycin D and harvested at 0, 3 and 6 h. RNA decay rate was determined to estimate the stability of LYPD1 (normalized to the expression at 0 h); **g** IGF2BP1 was knockdown in two HCC cells followed by the measurement of LYPD1 expression via qPCR; **h** RIP-qPCR validated that IGF2BP1 could bind to LYPD1 mRNA. Relative enrichment of LYPD1 mRNA in each group was showed with the normalization to input; **i** Rescue assays were employed to verify the impact of IGF2BP1 on ALKBH5-mediated modulation of LYPD1

Our MeRIP-seq analysis suggested that m^6^A peak of LYPD1 in 3’UTR shrank remarkably with the overexpression of ALKBH5 (Fig. 5a). To substantiate this result, we first conducted RIP assays using the anti-ALKBH5 antibody in Huh7 and HCCLM3 cells. We observed that ALKBH5 could enrich LYPD1 mRNA (Fig. 5b), implying that LYPD1 may be regulated in RNA level upon interaction with ALKBH5. Then MeRIP-qPCR assays with specific primers aiming at potential m^6^A sites revealed that knockdown of ALKBH5 could promote m^6^A modification of LYPD1 in 3’UTR, while activation of ALKBH5 led to a decreased m^6^A level in this site (Fig. 5c). To further demonstrate the essential role of m^6^A in the regulation of LYPD1, we designed a luciferase reporter inserting a wild-type (WT) LYPD1–3’UTR sequence or mutant (Mut) counterpart whose putative m^6^A sites were mutated (Fig. 5d). As expected, the luciferase activity of cells transfected with LYPD1-WT plasmid tended to increase when ALKBH5 was silenced, while that of mutant group seemed to be unaffected. And analogous results could be verified in ALKBH5-overexpressing cells (Fig. 5e). In addition, we found that ALKBH5 deficiency induced a slower degradation rate of LYPD1 mRNA, whereas ALKBH5 overexpression abolished the stability of LYPD1 (Fig. 5f).

Now that “readers” were crucially responsible for the direct effect on m^6^A-modified transcripts, we investigated potential effectors participating in the process illustrated above. As YTHDFs and IGF2BPs were extensively involved in the modulation of RNA stability [11], YTHDF1–2 and IGF2BP1–3 were knockdown successively in two HCC cells to examine the alterations of LYPD1 expression. We noticed that intervene of YTHDF1/2 and IGF2BP2/3 hardly impacted LYPD1 expression (Additional file 7: Figure S3f-i). Nevertheless, LYPD1 was significantly inhibited when IGF2BP1 was impaired (Fig. 5g), which was consistent with the knowledge that IGF2BP1 intended to promote the transcription of its targets [10]. And the interaction between IGF2BP1 protein and LYPD1 mRNA was confirmed by RIP assays (Fig. 5h). Moreover, knockdown of IGF2BP1 counteracted the accumulation of LYPD1 caused by ALKBH5 loss (Fig. 5i). In summary, LYPD1 is governed by ALKBH5-mediated m^6^A modification and recognized by IGF2BP1 which enhances its stability.

### LYPD1 is identified as an oncogenic driver in HCC

**Fig. 6:**
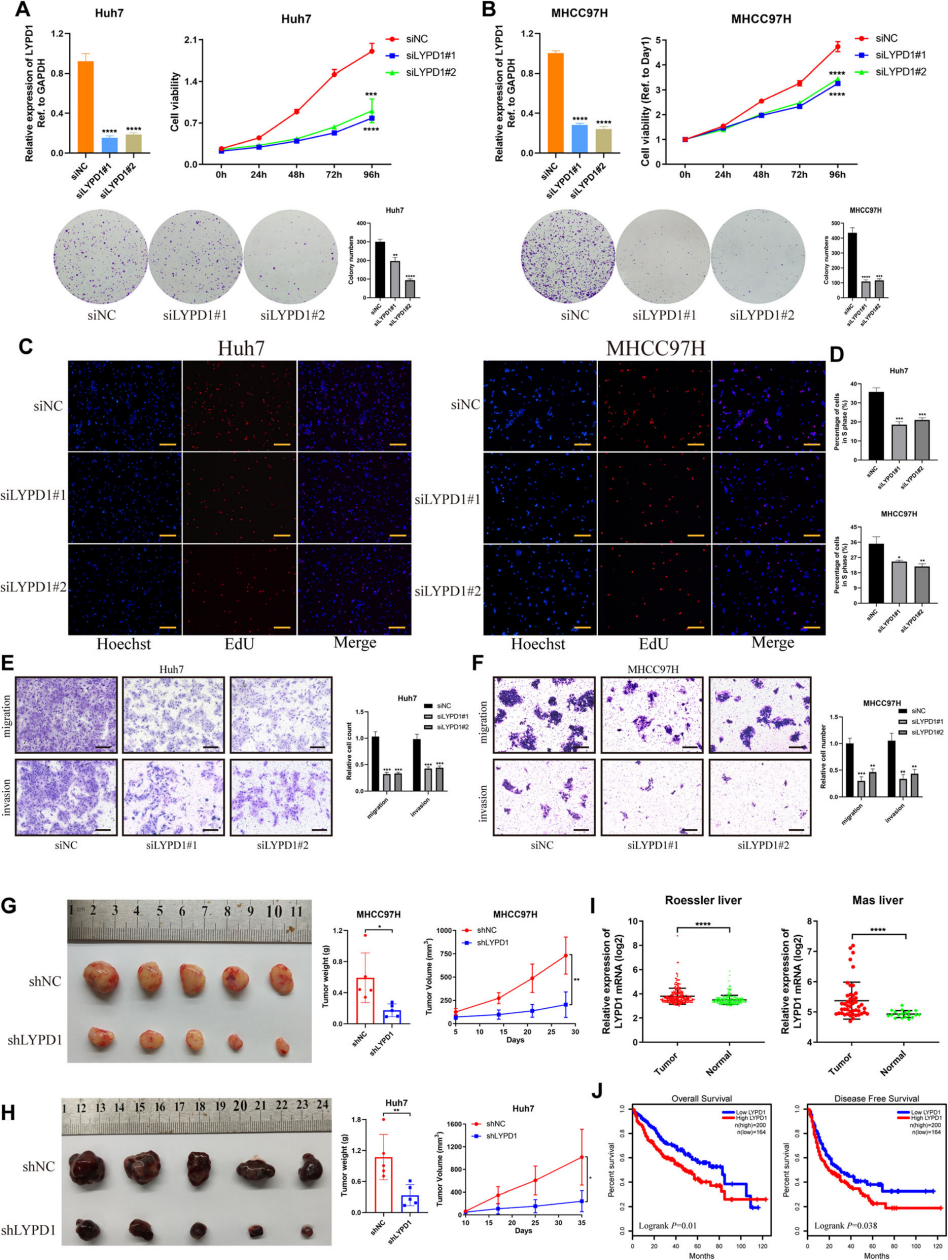
LYPD1 accelerates the malignant progression of HCC. **a** and **b** Knockdown of LYPD1 with two siRNAs was validated (left in upper panel) and proliferation abilities of LYPD1-silenced Huh7 (**a**) and MHCC97H (**b**) cells were determined using CCK-8 (right in upper panel) and colony formation assays (lower panel); **c** and **d** EdU assays were performed to detect the percent of cells with active DNA replication (scale bars in **c**, 200 μm); Hoechst staining showed the total cells, while EdU staining represented cells in S phase. And quantification data for each group (**d**) was displayed on the right; **e** and **f** Migration and invasion capabilities of Huh7 (**e**) and MHCC97H (**f**) cells after LYPD1 silencing were evaluated. Representative images (scale bars, 200 μm, left panel) and quantification charts (right panel) were shown; **g** and **h** Subcutaneous tumor models were established using stable LYPD1-knockdown Huh7 (**g**, *n* = 5) and MHCC97H (**h**, *n* = 5) cells. Photographs of tumors collected from mice were shown (left panel). Then tumor weights (middle panel) and growth curves (right panel) were exhibited to compare the difference of two groups. **i** GEO data analysis of HCC cohorts uploaded by Roessler et al. (GSE14520) and Mas et al. (GSE14323) showed the differential expression of LYPD1 in tumor and normal tissues; **j** Survival analysis of HCC patients based on expression of LYPD1 was conducted using TCGA data (*n* = 364)

To illustrate the role of LYPD1 in HCC, we established LYPD1-knockdown Huh7 and MHCC97H cell lines (Fig. 6a, b; Additional file 8: Figure S4a). CCK-8 and colony formation assays indicated that silencing of LYPD1 suppressed cell growth and viability (Fig. 6a, b), which was consistent with results of EdU (Fig. 6c, d). Moreover, loss of LYPD1 led to the inhibition of migration and invasion abilities of HCC cells (Fig. 6e, f). To evaluate the role of LYPD1 in vivo, lentiviruses carrying shRNA targeting at LYPD1 were transfected into Huh7 and MHCC97H cells with verified efficiency (Additional file 8: Figure S4b). Subsequently, subcutaneous implantation experiments were conducted in nude mice. As expected, knockdown of LYPD1 markedly impaired the growth of xenografted tumors (Fig. 6g, h).

Furthermore, bioinformatics analysis was carried out to explore the clinical association of LYPD1. The analysis of HCC cohort from TCGA and three other cohorts from Gene Expression Omnibus (GEO) datasets demonstrated that LYPD1 was up-regulated in tumorous tissues compared with normal tissues (Fig. 6i; Additional file 8: Figure S4c, d). Moreover, the up-regulation of LYPD1 was frequently detected in HCC patients who suffered nodal metastasis or belonged to higher tumor grades/stages (Additional file 8: Figure S4e-g). And pan-cancer analysis manifested that expression of LYPD1 was widely elevated across numerous cancers (Additional file 8: Figure S4h). Besides, Kaplan-Meier analysis implied higher LYPD1 expression correlated with poorer OS and disease-free survival (DFS) in HCC (Fig. 6j). Taken together, LYPD1 is activated during HCC development and promotes the oncogenesis of HCC.

### The effects of ALKBH5 inhibition are reversed by loss of LYPD1

**Fig. 7:**
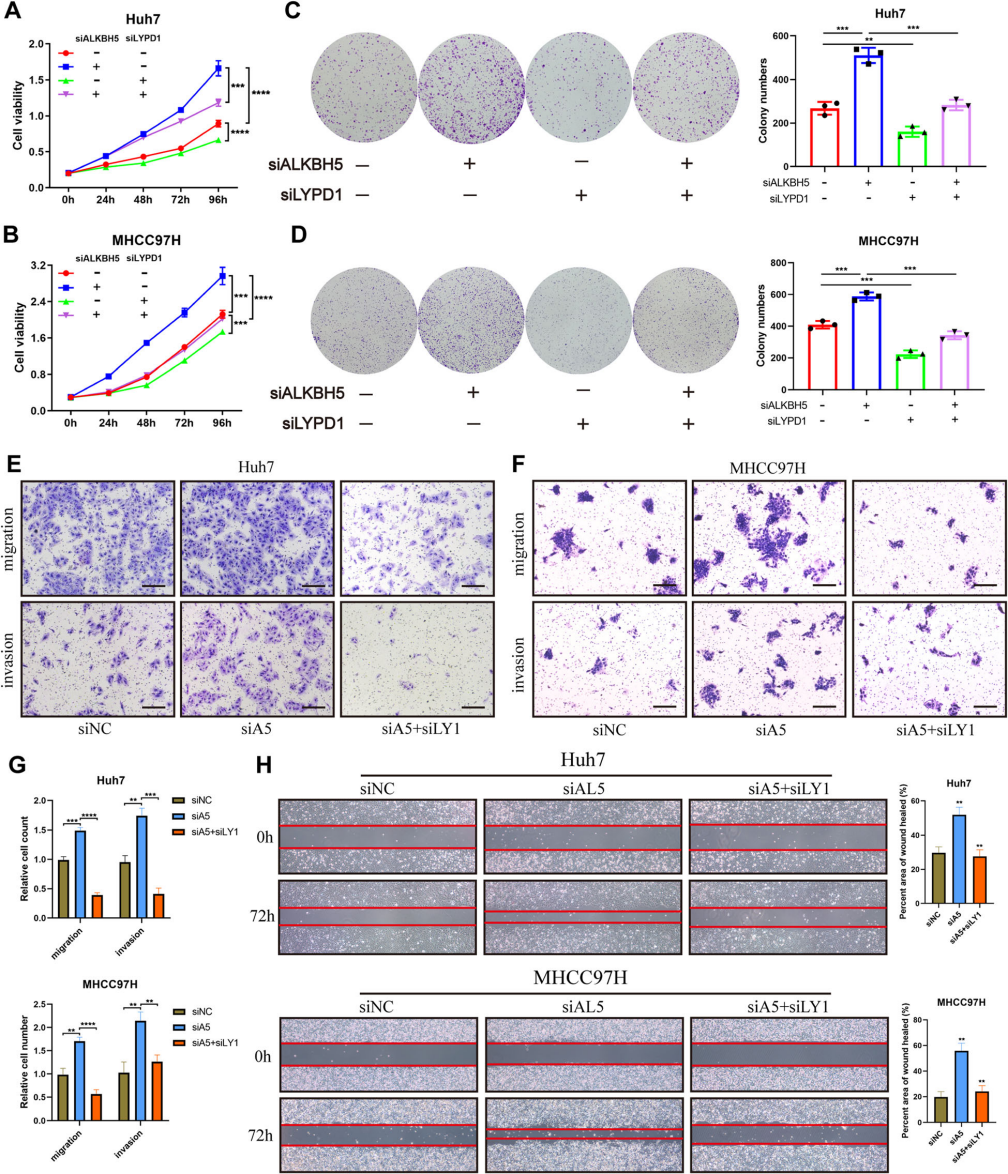
Dysregulation of the ALKBH5-LYPD1 axis triggers HCC malignancy. **a** and **b** CCK-8 proliferation assays were conducted in either ALKBH5-knockdown or LYPD1-knockdown Huh7 (**a**) and MHCC97H (**b**) cells; **c** and **d** Colony formation assays were carried out in either ALKBH5-silenced or LYPD1-silenced Huh7 (**c**) and MHCC97H (**d**) cells. Column diagrams (right panel) showed colony numbers of each group; **e**, **f** and **g** Representative images of transwell assays to examine the effects of LYPD1 knockdown on ALKBH5-silenced Huh7 (**e**) and MHCC97H (**f**) cells were shown (scale bars, 200 μm); Quantification data presented the relative count (refer to negative control group) of cells which passed through the chamber membrane (**g**); **h** Representative images of wound healing assays conducted in ALKBH5/LYPD1-rescued cells were shown (left panel). And percent area of wound healed in each group was quantified (right panel)

To confirm that the observed phenotypes were mediated by the dysregulation of ALKBH5-LYPD1 axis, we conducted several functional rescue assays. As CCK-8 and colony assays showed, knockdown of ALKBH5 led to the enhanced proliferation capacity in two HCC cells, which could be reverted by LYPD1 silencing (Fig. 7a-d). Knockdown of LYPD1 also significantly abolished the increased mobility ability induced by ALKBH5 loss (Fig. 7e-g). Besides, wound healing assays suggested that the inhibition of ALKBH5 failed to promote cell migration in LYPD1-silenced Huh7 and MHCC97H cells (Fig. 7h). To sum up, dysfunction of LYPD1 may account for the ALKBH5-mediated proliferation or mobility signatures of HCC cells.

### Clinical relevance of the ALKBH5/LYPD1 axis in HCC

**Fig. 8:**
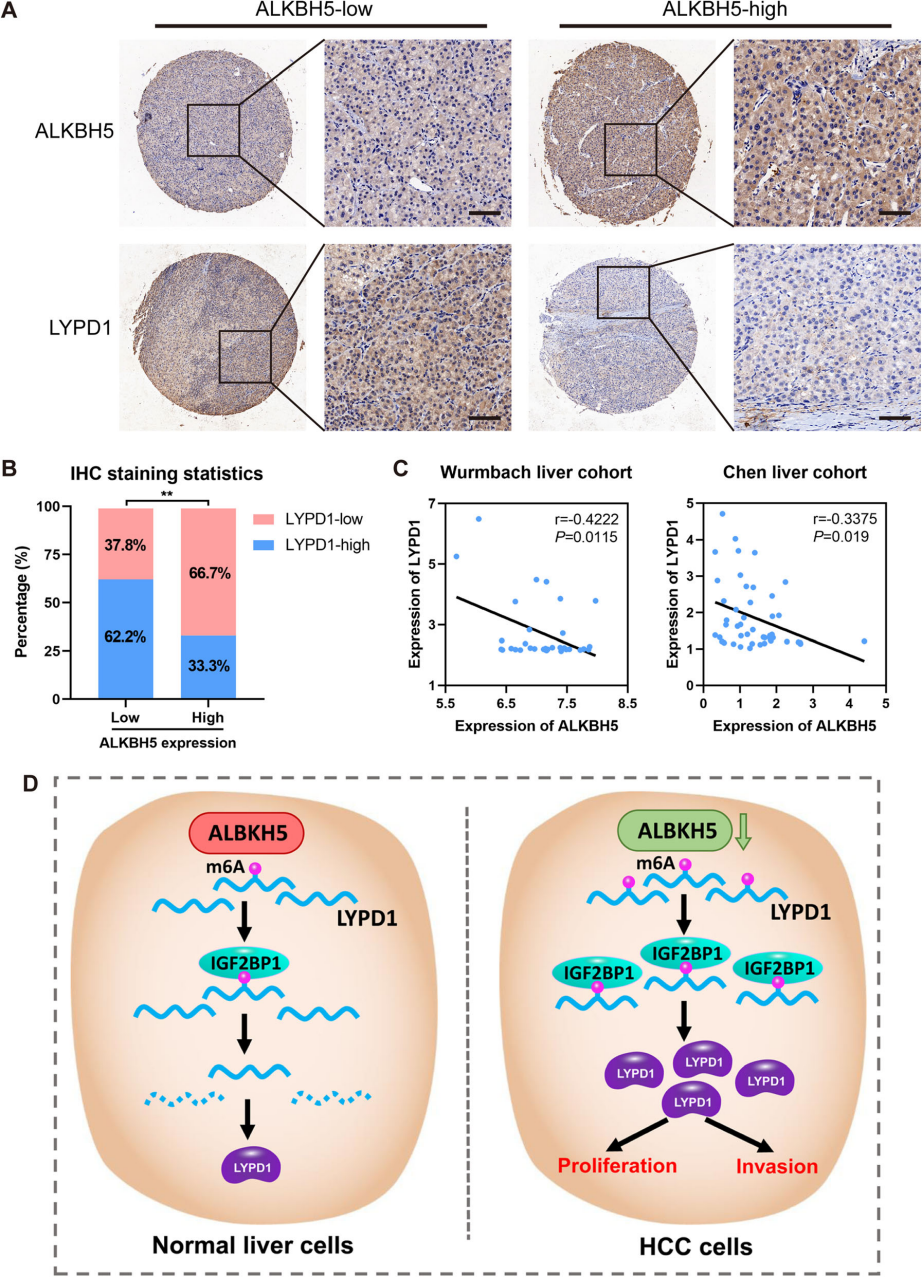
Low ALKBH5 expression is interrelated with high LYPD1 expression in HCC. **a** The TMA cohort (cohort two) was subject to IHC staining for both ALKBH5 and LYPD1. Representative images of higher or lower ALKBH5 staining and corresponding LYPD1 staining were shown, respectively (scale bars, 50 μm; magnification, 100× and 400×); **b** IHC staining statistics showed the percentage of HCC samples displaying higher or lower ALKBH5 levels and corresponding LYPD1 expression. For the same specimens, the IHC intensity of ALKBH5 and LYPD1 were frequently negatively correlated. **c** GEO data analysis of two HCC cohorts (GSE6764 and GSE3500) showed the inverse correlation of ALKBH5 and LYPD1 based on RNA expression; **d** A schematic illustration was proposed to summarize our findings about ALKHB5-guided m^6^A modulation on LYPD1 (the green and red colors indicated the activated and inhibited status, respectively). In brief, ALKBH5 is down-regulated in HCC cells compared with normal liver cells. Deficiency of ALKBH5 leads to an elevated m^6^A level of LYPD1 which is recognized and strengthened by the m^6^A effector IGF2BP1, thus reinforcing the expression of LYPD1. Accumulated LYPD1 promotes the proliferation and invasion capabilities of HCC cells, and further drives the tumorigenesis of HCC

To further explore the correlation between expression of ALKBH5 and LYPD1 in HCC tissues, IHC staining of these two proteins were performed on TMA from the second cohort. As expected, approximately 62.2% of specimens with lower expression of ALKBH5 presented stronger LYPD1 staining, while nearly 66.7% of those with higher ALKBH5 expression exhibited weaker LYPD1 dyeing (Fig. 8a, b). Furthermore, the analysis of two independent GEO datasets revealed that ALKBH5 negatively interrelated with LYPD1 in RNA level (Fig. 8c). In conclusion, the expression of ALKBH5 and LYPD1 are inversely correlated in HCC samples.

## Discussion

Accumulating evidence demonstrates that the aberration of m^6^A modification is dramatically involved in the pathogenesis of multiple diseases including HCC [27]. Actually, our previous work focuses on one of the m^6^A methyltransferases WTAP, which is identified to promote the progression of HCC in an m^6^A-dependent manner [18]. In addition, METTL3, METTL14 and KIAA1429 have been successively reported to impact the growth and invasion of HCC cells via diverse mechanisms [16, 17, 19]. And two studies have reported ambiguous results about FTO, which may serve as either an oncogene or a tumor suppressor in HCC [21, 22]. Meanwhile, our preliminary experiments also found the controversial effects of FTO on proliferation abilities in different HCC cells (Additional file 9: Figure S5a-k). It implies that roles of FTO in HCC may be momentous but perplexing. However, whether ALKBH5 contributes to the evolution of HCC still remains obscure. Therefore, our present study concentrated on the role of ALKBH5, and first addressed that the decreased ALKBH5 expression correlated with worse survival in HCC patients. We functionally confirmed that ALKBH5 suppressed growth and invasion abilities of HCC cells in vitro and in vivo. Mechanistically, LYPD1 was regulated by ALKBH5 via an m^6^A-mediated and IGF2BP1-associated pattern. And LYPD1 was subsequently verified as an oncogenic driver in HCC. Altogether, the dysregulation of ALKBH5/LYPD1 axis facilitated the progression of HCC (Fig. 8d).

ALKBH5 is a nucleic acid oxygenase which can catalyze the demethylation of m^6^A-labelled RNA [9]. Initially, it is reported that ALKBH5 deficiency leads to impaired fertility by controlling splicing of long 3’UTR mRNA in germ cells [9, 28]. And following researches in the field of viral infection reveal that ALKBH5 is involved in antiviral processes via m^6^A-guided regulation on cellular metabolism and innate immunity [29, 30]. Besides, Song et al. establish the link between ALKBH5 and autophagy in ischemic heart diseases [31]. Furthermore, ALKBH5 plays an essential part in the tumorigenesis as well. ALKBH5 enhances self-renewal and oncogenesis of glioblastoma by sustaining FOXM1 expression [32], and it also mediates the hypoxia-induced stem cell phenotypes of breast cancer [33]. However, Tang et al. uncover the tumor suppressive function of ALKBH5 through m^6^A-dependent inactivation of Wnt signaling [34]. In addition, ALKBH5 inhibits tumor growth and metastasis via abolishing expression and activity of YAP in non-small cell lung cancer [35]. And our findings also clarify that ALKBH5 inhibits HCC malignancy through impairing the expression of LYPD1. It implies that effects of ALKBH5 on carcinogenesis depend on the specific tissue context and different downstream molecules.

It is noteworthy that although downstream targets of ALKBH5 have been frequently explored as mentioned above, upstream masters of ALKBH5 are still vague. Hence, the factors which may induce ALKBH5 deficiency in HCC merit a debate. Based on TCGA pan-cancer data, Li et al. evaluate the alteration frequency of copy number variations (CNVs) for all m^6^A regulators and demonstrate that ALKBH5 gains prevalent CNV deletions in HCC (frequency of 0.432) [36]. CNV loss may be partly responsible for down-regulation of ALKBH5. In addition, microRNAs (miRNA) have been shown to inhibit several m^6^A enzymes [37]. We find miR-17-3p, which promotes the growth and invasion of HCC cells [38], is the potential miRNA targeting at ALKBH5 as predicted by TarBase and TargetScan tools. It should be further validated whether miR-17-3p or other miRNAs can regulate the level of ALKBH5. And it is reported that cigarette smoke condensate may induce the hypomethylation of ALKBH5 CpG island in esophageal squamous cell carcinoma [39]. That suggests that ALKBH5 can also be regulated by DNA methylation, which is widely involved in HCC pathogenesis. Besides, histone acetylation and methylation actively participate in the modulation of m^6^A enzymes as well [40]. Whether ALKBH5 is controlled by suppressive histone modifications such as H3K9me3 or H3K27me3 requires further investigations.

Although LYPD1 is identified as the target of ALKBH5, we wonder whether another m^6^A demethylase FTO is also responsible for the demethylation of LYPD1. To address this crucial issue, the alterations of LYPD1 expression upon the silencing of FTO were firstly checked. We found that loss of FTO seemed to impact little on the level of LYPD1 (Additional file 10: Figure S6a, b). Then MeRIP-qPCR assays revealed that knockdown of FTO did not affect the m^6^A status of LYPD1, while ALKBH5 deficiency significantly triggered an elevated m^6^A level. And there was no difference between single ALKBH5-silencing group and double ALKBH5/FTO-silencing group (Additional file 10: Figure S6c-g). These results demonstrate that m^6^A modification of LYPD1 may be selectively governed by ALKBH5, instead of FTO, which is also supported by the outcomes of luciferase reporter assays (Additional file 10: Figure S6h, i).

Actually, LYPD1 was predicted as a sort of glycosylphosphatidylinositol (GPI)-anchored and membrane-bound protein [41]. It was originally identified from central nervous systems [42] and it may govern anxiety by binding to neuronal nicotinic acetylcholine receptors (nAChRs) [43]. Albeit LYPD1 was judged as a tumor suppressor in HeLaHF cells [44], few other investigations offered information about its role in cancer. In our study, loss of LYPD1 disrupted the proliferation ability and invasion potential of HCC cells (Fig. 6a-h), while LYPD1 expression was elevated in tumor tissues and high level of LYPD1 indicated a poorer prognosis of HCC (Fig. 6i, j and Additional file 8: Figure S4c-g). Here we have systematically illustrate the cancer-related behavior of LYPD1 and its upstream partners within the m^6^A-based modulation, providing novel insights into functions of LYPD1 in tumorigenesis. Perhaps we should further exploit how to make it feasible to abolish LYPD1 activity clinically.

Although we have supplied abundant evidence to support the significant role of m^6^A-regulated ALKBH5/LYPD1 axis in HCC progression, there are still several drawbacks in our work. For example, the results of ALKBH5/IGF2BP1 rescue assays showed a little difference between two HCC cells, which implies that IGF2BP1 may not be the only m^6^A reader downstream of ALKBH5 loss (Fig. 5i). Although we have screened YTHDFs and IGF2BPs family which are closely related to RNA stability modulation (Additional file 7: Figure S3f-i), other effector proteins such as YTHDCs (involved in alternative splicing or nuclear export) [45, 46], HNRNPs (related to molecular structure) [47] and other non-canonical readers (like HuR, etc.) have not been investigated detailedly. Maybe some of them can also participate in the regulation of LYPD1, which deserves a further exploration.

Besides, the underlying mechanisms of LYPD1-mediated modulation of downstream pathways is not fully characterized. To hunt for some clues, we have ever re-analyzed the results of transcriptome sequencing. Gene ontology analysis demonstrated that ALKBH5 expression was tightly correlated with cell motility and proliferation (Additional file 10: Figure S6j), which is consistent with our functional results. In addition, ALKBH5 may be involved in the regulation of PI3K and GTPases pathways (Additional file 10: Figure S6k). The PI3K/AKT/mTOR cascade is one of the most crucial signaling in tumor, which controls various cellular activities including cell growth and migration [48]. And Rho GTPases are responsible for the domination of cytoskeleton organization and cell mobility [49]. Our western blotting outcomes revealed that knockdown of ALKBH5 efficiently triggered the PI3K/AKT/mTOR and Rho GTPases pathways, while suppression of LYPD1 retrieved these activated machineries (Additional file 10: Figure S6l). To ensure that these two pathways were explicitly involved in ALKBH5/LYPD1-dependent modulation, further work was required including exploring direct link between LYPD1 and downstream signaling and assessing whether ALKBH5/LYPD1 axis could remodel the sensitivity of HCC cells to inhibitors of PI3K/AKT/mTOR or GTPases pathways in vitro and in vivo.

## Conclusion

In summary, our work has revealed the tumor suppressor properties of ALKBH5 in HCC development. Down-regulation of ALKBH5 activates the m^6^A machinery contributing to the epigenetic activation of LYPD1 which is recognized and stabilized by IGF2BP1. Our findings highlight the attractive values of m^6^A demethylases and enrich the understanding of m^6^A epitranscriptomic modification in cancer research, further providing novel insights into exploiting effective predictors and therapeutic strategies for HCC.

## Supplementary information

**Additional file 1 **: **Table S1.** Clinical characteristics of 90 HCC patients depending on ALKBH5 expression.

**Additional file 2 **: **Table S2.** Target sequences of siRNAs and shRNAs utilized in this work.

**Additional file 3 **: **Table S3.** Sequences of primers utilized in this study.

**Additional file 4 **: **Table S4.** Antibodies utilized in this work.

**Additional file 5 **: **Figure S1.** Clinical significance and transfection efficiency of ALKBH5. **a** Kaplan-Meier analysis of all HCC patients based on ALKBH5 expression (from TCGA cohort, analyzed with KM plotter, https://kmplot.com/analysis/); **b** Kaplan-Meier analysis of HCC patients with early stages (stage 1 and 2, from TCGA cohort) based on ALKBH5 expression; **c** and **d** Protein (**c**) and RNA (**d**) expression of ALKBH5 in HCC cell lines; **e**, **f**, **g** and **h** The transient knockdown and stable overexpression efficiency of ALKBH5 in three HCC cells was determined by western blotting (**e**) and qPCR (**f-h**); **i**, **j** and **k** The stable knockdown efficiency of ALKBH5 was measured via western blotting (**i**) and qPCR (**j**, **k**). OS: overall survival; PFS: progression-free survival.

**Additional file 6 **: **Figure S2.** Further in vitro and in vivo information about the roles of ALKBH5 in HCC cells. **a** and **b** The knockdown and re-expression efficiency of ALKBH5 in two HCC cells were determined via qPCR (**a**) and western blotting (**b**); **c** and **d** CCK-8 (upper panel) and colony assays (lower panel) were conducted to check the effects of ALKBH5 re-expression in ALKBH5-silenced in Huh7 (**c**) and MHCC97H (**d**) cells. **e**, **f** and **g** EdU assays were employed to further determine the effects of ALKBH5 reactivation on ALKBH5-knockdown Huh7 (**e**) and MHCC97H (**f**) cells. And percentage of cells in S phase was exhibited (**g**). **h** Typical IHC images of subcutaneous tumors using ALKBH5-overexpressed or vector transfected HCCLM3 cells were shown (scale bars: 50 μm); Staining of ALKBH5 was applied to validate the transfection efficiency, while intensity of PCNA staining represented the proliferation capability of tumors. **i** Representative HE staining images of metastasis in lungs induced by tail vein injection of negative control or ALKBH5-silenced MHCC97H cells were presented; **j** Tumors of xenografted mice implanted with ALKBH5-overexpressed or control HCCLM3 cells were subject to RNA isolation. The m^6^A level of each group was measured using m^6^A dot blot assays. And the representative images of dot blots were shown.

**Additional file 7 **: **Figure S3.** Screening of ALKBH5 targets and potential m^6^A effectors of LYPD1. **a, b**, **c**, **d** and **e** Expression of COLCA2 (**a**), TMED7 (**b**), CYP4F3 (**c**), IL17RB (**d**) and VCAN (**e**) were checked in ALKBH5-knockdown or -overexpressed cells, respectively. Expression of ABCA4 was too low to detect, thus its data was not shown; **f** LYPD1 was measured by qPCR after YTHDF1 was knockdown in Huh7 and MHCC97H cells; **g** LYPD1 was determined by qPCR after YTHDF2 was knockdown in HCC cells; **h** LYPD1 was determined using qPCR when IGF2BP2 was knockdown in HCC cells; **i** LYPD1 was measured using qPCR after IGF2BP3 was knockdown in HCC cells.

**Additional file 8 **: **Figure S4.** LYPD1 was up-regulated in HCC. **a** Knockdown efficiency of LYPD1 using siRNA was verified in Huh7 and MHCC97H cells by western blotting; **b** Knockdown efficiency of LYPD1 using shRNA was confirmed via qPCR; **c** and **d** Expression of LYPD1 in HCC patients from TCGA (**c**) or GEO (**d**, GSE6764) data was shown; **e**, **f** and **g** Expression of LYPD1 in HCC cohorts based on TCGA data stratified by nodal metastasis status (**e**), tumor grade (**f**) and tumor stage (**g**). (**e** and **f**: analyzed by UALCAN; **g**: analyzed by GEPIA) **h**. Pan-cancer atlas of LYPD1 expression in HCC samples (data from TCGA, analyzed by UALCAN; blue color represented normal group and red color represented tumor group).

**Additional file 9 **: **Figure S5.** Controversial functional roles of FTO in different HCC cells. **a** and **b** Knockdown efficiency of FTO in Huh7 and MHCC97H were measured by western blotting (**a**) and qPCR (**b**); **c** and **d** CCK-8 and colony formation assays were conducted in FTO-silenced Huh7 (**c**) and MHCC97H (**d**) cells. Column charts showed colony numbers of each group (right panel). Loss of FTO contributed little to the proliferation abilities of these two cells. **e, f** and **g** Negative control and FTO-silenced Huh7 (**e**) or MHCC97H (**f**) cells were subject to EdU assays. Percentage of cells in S phase was quantified in column charts (**g**); **h** and **i** Knockdown efficiency of FTO in HepG2 and Hep3B were determined by western blotting (**h**) and qPCR (**i**); **j** and **k** CCK-8 and colony formation assays were conducted in FTO-knockdown HepG2 (**j**) and Hep3B (**k**) cells. Surprisingly, inhibition of FTO suppressed the proliferation capabilities of these two cells.

**Additional file 10 **: **Figure S6.** The explorations of whether FTO can regulate the m^6^A modification of LYPD1 and possible downstream pathways of ALKBH5/LYPD1 axis. **a** and **b** Expression of LYPD1 was measured when FTO was silenced in Huh7 and MHCC97H cells using western blotting (**a**) and qPCR (**b**) assays; **c** Transfection efficiency was measured by western blotting assays in two HCC cells with individual or double knockdown of FTO and ALKBH5; **d** and **e** Transfection efficiency was determined via qPCR assays in Huh7 (**d**) and MHCC97H (**e**) cells with individual or double knockdown of FTO and ALKBH5; **f** and **g** Relative m^6^A enrichment of LYPD1 in Huh7 (**f**) and MHCC97H (**g**) cells with single or double knockdown of FTO and ALKBH5 were determined by MeRIP-qPCR assays. **h** and **i** Relative luciferase activity of Huh7 (**h**) and MHCC97H (**i**) cells transfected with the LYPD1-wild type or LYPD1-m^6^A sites-mutated construct were measured. For each group, FTO and ALKBH5 are individually or double knockdown. **j** and **k** GO functional categories containing BP (**j**) and MF (**k**) of RNA sequencing using ALKBH5-overexpression or control HCCLM3 cells. When ALKBH5 was overexpressed, those down-regulated transcripts were prominently enriched in BP including epithelial cell migration, cell proliferation or cell adhesion. Meanwhile, they were enriched in MF which mainly contains PI3K activity and GTPase regulator activity; **l** Western blotting analyses were performed in ALKBH5-silenced HCC cells to check the impacts of ALKBH5 on PI3K-AKT-mTOR and Rho GTPases signaling. Then effects of following LYPD1 inhibition on these pathways were examined. Members of PI3K-AKT-mTOR (including p85, p110, p-AKT, p-mTOR, p-70S6K, p-RPS6) and Rho GTPases (including CDC42, RhoA, RhoC and Rac1/2/3) pathways were tested, respectively. BP: biological processes; MF: molecular function.

**Additional file 11.** STR certificates for Huh7 (page 2–4), HCCLM3 (page 5–7) and MHCC97H (page 8–17) cell lines.

**Additional file 12.** Major R codes for MeRIP-seq analyses.

## Data Availability

All data created and analyzed during this current work are involved in this published article (and its supplementary information files) or available on published databases (TCGA or GEO). And GEO accession number of our sequencing is GSE149510.

## Abbreviations

HCC: Hepatocellular carcinoma
m^6^A: N6-methyladenosine
ALKBH5: AlkB homolog 5
MeRIP-seq: Methylated RNA immunoprecipitation sequencing
RNA-seq: RNA sequencing
RIP: RNA immunoprecipitation
LYPD1: LY6/PLAUR Domain Containing 1
IGF2BP1: Insulin like Growth Factor 2 mRNA Binding Protein 1
TMA: Tissue microarrays
TCGA: The Cancer Genome Atlas
GEO: Gene Expression Omnibus
qPCR: Quantitative real-time PCR
IHC: Immunohistochemistry
CCK-8: Cell Counting Kit-8
EdU: 5-ethynyl-20-deoxyuridine
OS: Overall survival
RFS: Recurrence-free survival
DFS: Disease-free survival
PI3K: 1-Phosphatidylinositol-3-Kinase
IGV: Integrative genomics viewer
CI: Confidence interval
HR: Hazard ratio
DAPI: 4′,6-diamidino-2-phenylindole
