# Supplementary material for: ALKBH5 suppresses malignancy of hepatocellular carcinoma via m6A-guided epigenetic inhibition of LYPD1
Source: Mol Cancer. 2020 Aug 10;19:123. doi: 10.1186/s12943-020-01239-w (PMC7416417; doi:10.1186/s12943-020-01239-w)
Supplement: Supplementary file 1 — Additional file 1 : Table S1. Clinical characteristics of 90 HCC patients depending on ALKBH5 expression. [file 12943_2020_1239_MOESM1_ESM.docx]

| Table S1 Clinical characteristics of 90 HCC patients depending on ALKBH5 expression. | | | | | | |
| --- | --- | --- | --- | --- | --- | --- |
| Fetures | ALKBH5-high | ALKBH5-low | | n | *Χ^2^* | *P* |
| All cases | 45 | | 45 | 90 |  |  |
| Age(years) |  | |  |  | 0.278 | 0.598 |
| ≤60 | 37 | | 35 | 72 |  |  |
| ＞60 | 8 | | 10 | 18 |  |  |
| Gender |  | |  |  | 0.000 | >0.999 |
| Males | 37 | | 37 | 74 |  |  |
| Females | 8 | | 8 | 16 |  |  |
| Differentiation | | |  |  | 1.196 | 0.274 |
| Low | 31 | | 26 | 57 |  |  |
| High | 14 | | 19 | 33 |  |  |
| AJCC stage |  | |  |  | 1.272 | 0.259 |
| Stage I | 33 | | 28 | 61 |  |  |
| Stage II & III | 12 | | 17 | 29 |  |  |
| Cirrhosis nodules size | | |  |  | 2.880 | 0.897 |
| ≤3cm | 21 | | 29 | 50 |  |  |
| ＞3cm | 24 | | 16 | 40 |  |  |
| Tumor encapsulation |  | |  |  | 0.045 | 0.833 |
| Absent | 21 | | 22 | 43 |  |  |
| Present | 24 | | 23 | 47 |  |  |
| AFP |  | |  |  | 1.196 | 0.274 |
| ≤400 ug/L | 31 | | 26 | 57 |  |  |
| ＞400 ug/L | 14 | | 19 | 33 |  |  |
| Recurrence |  | |  |  |  |  |
| No | 22 | | 15 | 37 | 3.717 | 0.054 |
| Yes | 23 | | 30 | 53 |  |  |

Note: Pearson Chi-Square tests were applied to evaluate the association between categorical variables.
