# Supplementary figures and images for: ALKBH5 suppresses malignancy of hepatocellular carcinoma via m6A-guided epigenetic inhibition of LYPD1
Source: Mol Cancer. 2020 Aug 10;19:123. doi: 10.1186/s12943-020-01239-w (PMC7416417; doi:10.1186/s12943-020-01239-w)

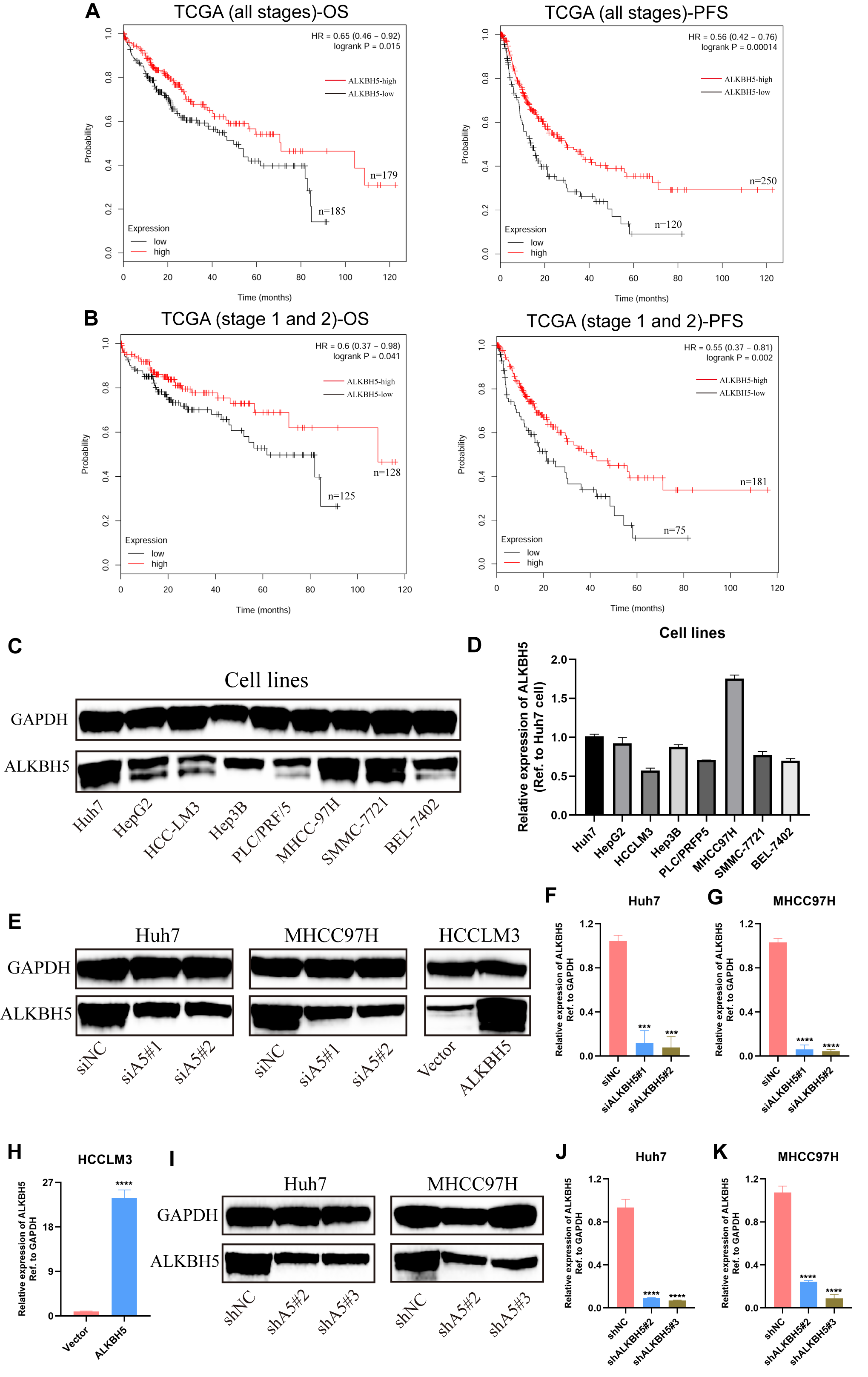

Supplement: Supplementary file 5 — Additional file 5 : Figure S1. Clinical significance and transfection efficiency of ALKBH5. a Kaplan-Meier analysis of all HCC patients based on ALKBH5 expression (from TCGA cohort, analyzed with KM plotter, https://kmplot.com/analysis/); b Kaplan-Meier analysis of HCC patients with early stages (stage 1 and 2, from TCGA cohort) based on ALKBH5 expression; c and d Protein (c) and RNA (d) expression of ALKBH5 in HCC cell lines; e, f, g and h The transient knockdown and stable overexpression efficiency of ALKBH5 in three HCC cells was determined by western blotting (e) and qPCR (f-h); i, j and k The stable knockdown efficiency of ALKBH5 was measured via western blotting (i) and qPCR (j, k). OS: overall survival; PFS: progression-free survival. [file 12943_2020_1239_MOESM5_ESM.tif]

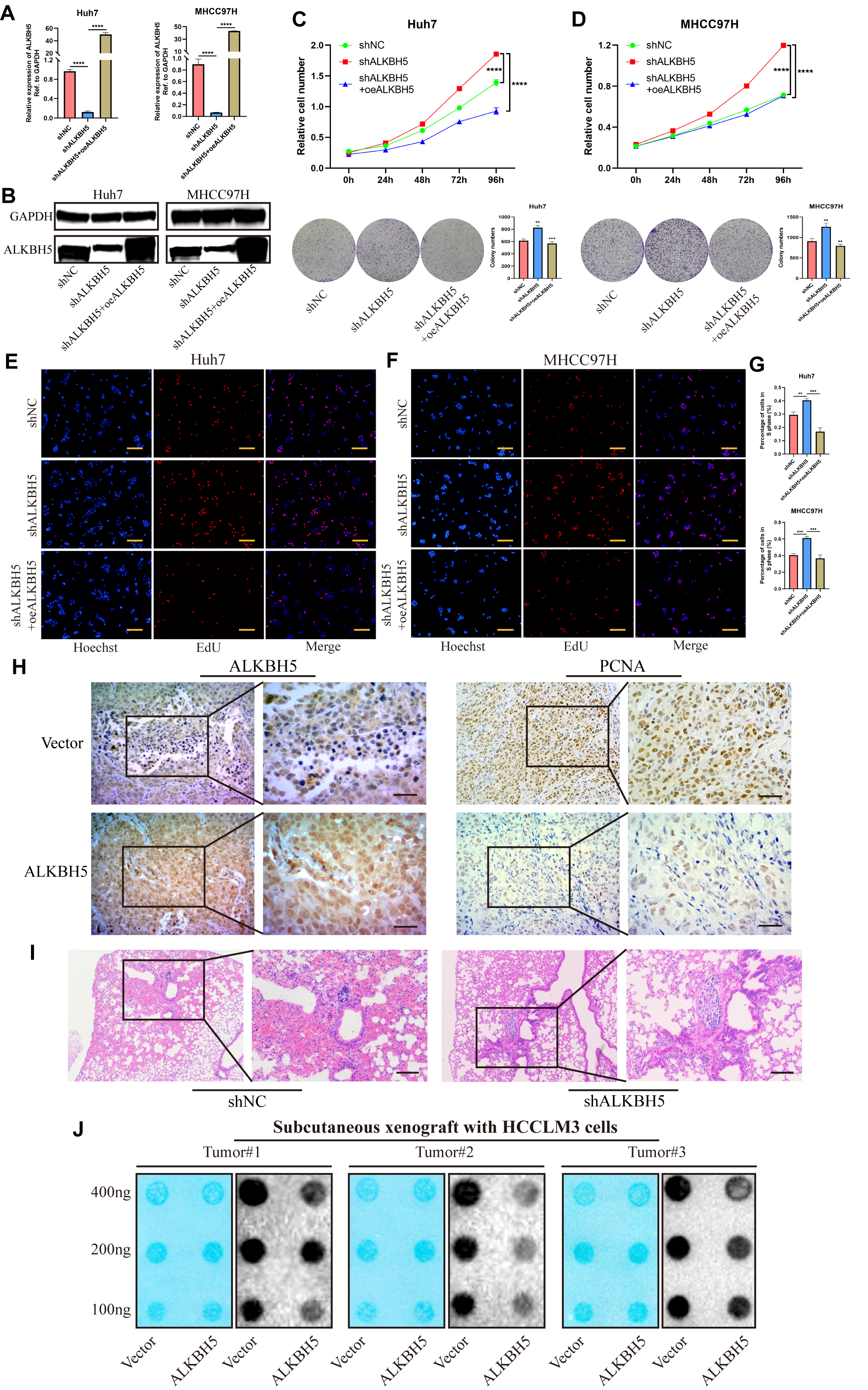

Supplement: Supplementary file 6 — Additional file 6 : Figure S2. Further in vitro and in vivo information about the roles of ALKBH5 in HCC cells. a and b The knockdown and re-expression efficiency of ALKBH5 in two HCC cells were determined via qPCR (a) and western blotting (b); c and d CCK-8 (upper panel) and colony assays (lower panel) were conducted to check the effects of ALKBH5 re-expression in ALKBH5-silenced in Huh7 (c) and MHCC97H (d) cells. e, f and g EdU assays were employed to further determine the effects of ALKBH5 reactivation on ALKBH5-knockdown Huh7 (e) and MHCC97H (f) cells. And percentage of cells in S phase was exhibited (g). h Typical IHC images of subcutaneous tumors using ALKBH5-overexpressed or vector transfected HCCLM3 cells were shown (scale bars: 50 μm); Staining of ALKBH5 was applied to validate the transfection efficiency, while intensity of PCNA staining represented the proliferation capability of tumors. i Representative HE staining images of metastasis in lungs induced by tail vein injection of negative control or ALKBH5-silenced MHCC97H cells were presented; j Tumors of xenografted mice implanted with ALKBH5-overexpressed or control HCCLM3 cells were subject to RNA isolation. The m6A level of each group was measured using m6A dot blot assays. And the representative images of dot blots were shown. [file 12943_2020_1239_MOESM6_ESM.tif]

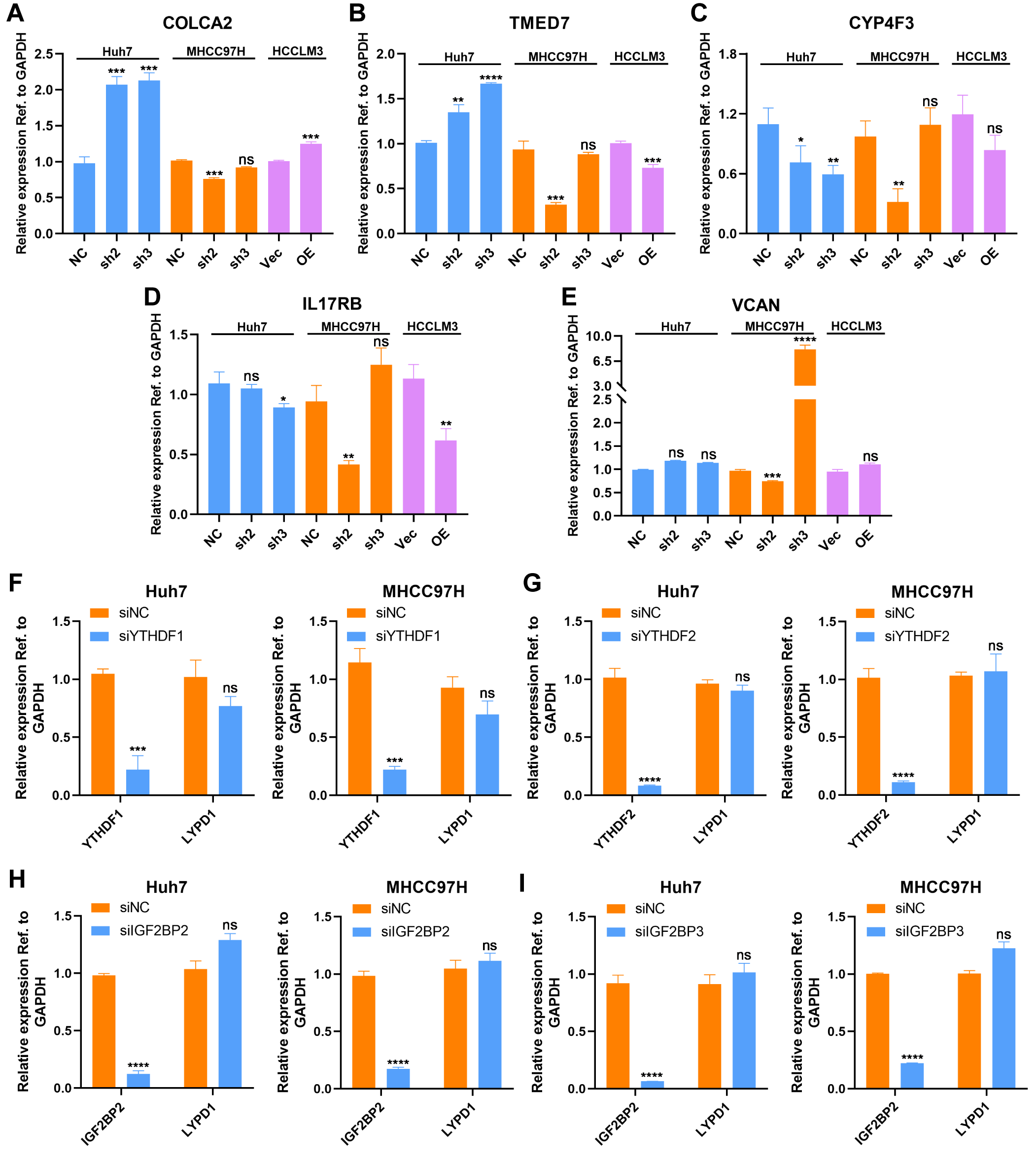

Supplement: Supplementary file 7 — Additional file 7 : Figure S3. Screening of ALKBH5 targets and potential m6A effectors of LYPD1. a, b, c, d and e Expression of COLCA2 (a), TMED7 (b), CYP4F3 (c), IL17RB (d) and VCAN (e) were checked in ALKBH5-knockdown or -overexpressed cells, respectively. Expression of ABCA4 was too low to detect, thus its data was not shown; f LYPD1 was measured by qPCR after YTHDF1 was knockdown in Huh7 and MHCC97H cells; g LYPD1 was determined by qPCR after YTHDF2 was knockdown in HCC cells; h LYPD1 was determined using qPCR when IGF2BP2 was knockdown in HCC cells; i LYPD1 was measured using qPCR after IGF2BP3 was knockdown in HCC cells. [file 12943_2020_1239_MOESM7_ESM.tif]

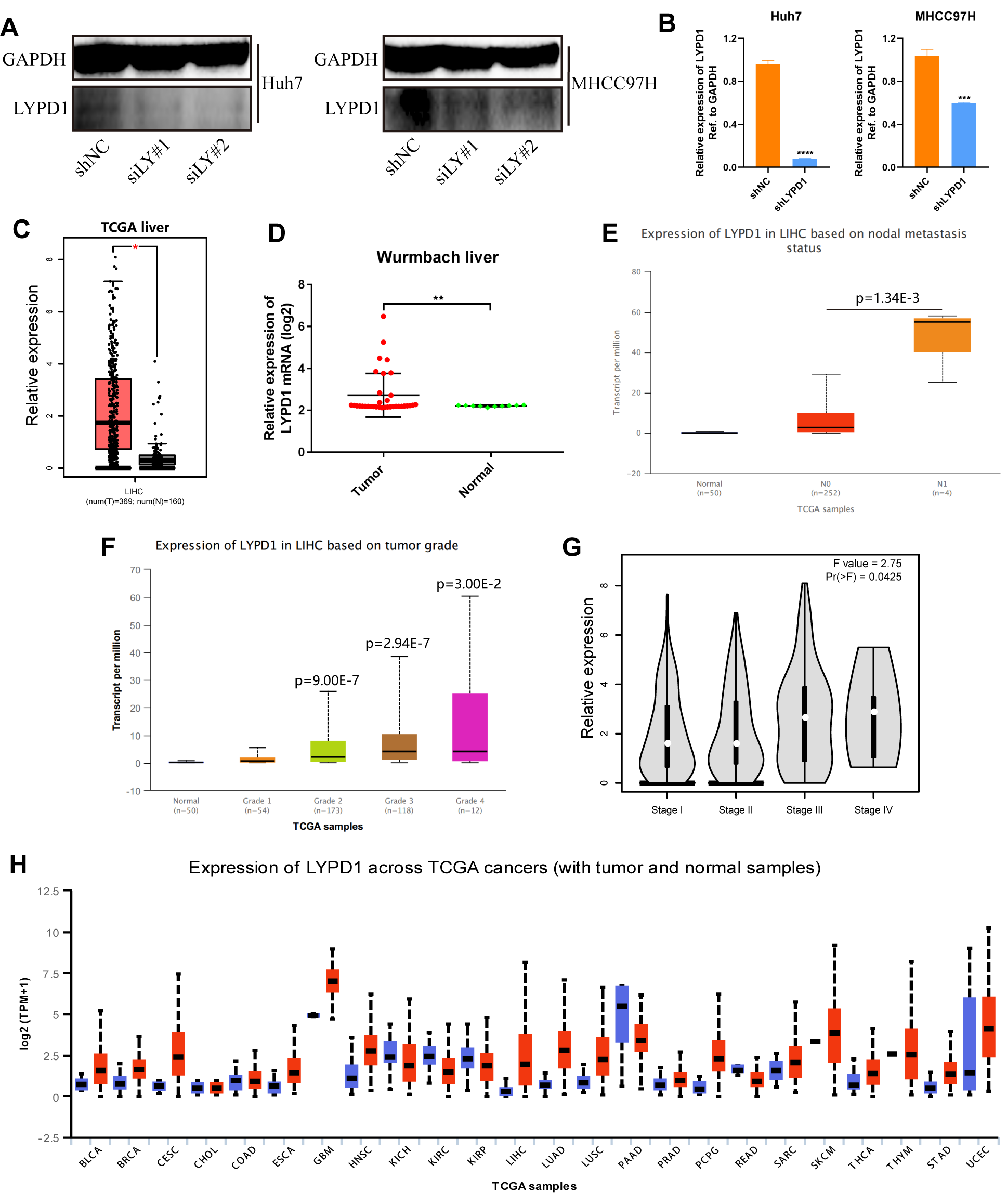

Supplement: Supplementary file 8 — Additional file 8 : Figure S4. LYPD1 was up-regulated in HCC. a Knockdown efficiency of LYPD1 using siRNA was verified in Huh7 and MHCC97H cells by western blotting; b Knockdown efficiency of LYPD1 using shRNA was confirmed via qPCR; c and d Expression of LYPD1 in HCC patients from TCGA (c) or GEO (d, GSE6764) data was shown; e, f and g Expression of LYPD1 in HCC cohorts based on TCGA data stratified by nodal metastasis status (e), tumor grade (f) and tumor stage (g). (e and f: analyzed by UALCAN; g: analyzed by GEPIA) h. Pan-cancer atlas of LYPD1 expression in HCC samples (data from TCGA, analyzed by UALCAN; blue color represented normal group and red color represented tumor group). [file 12943_2020_1239_MOESM8_ESM.tif]

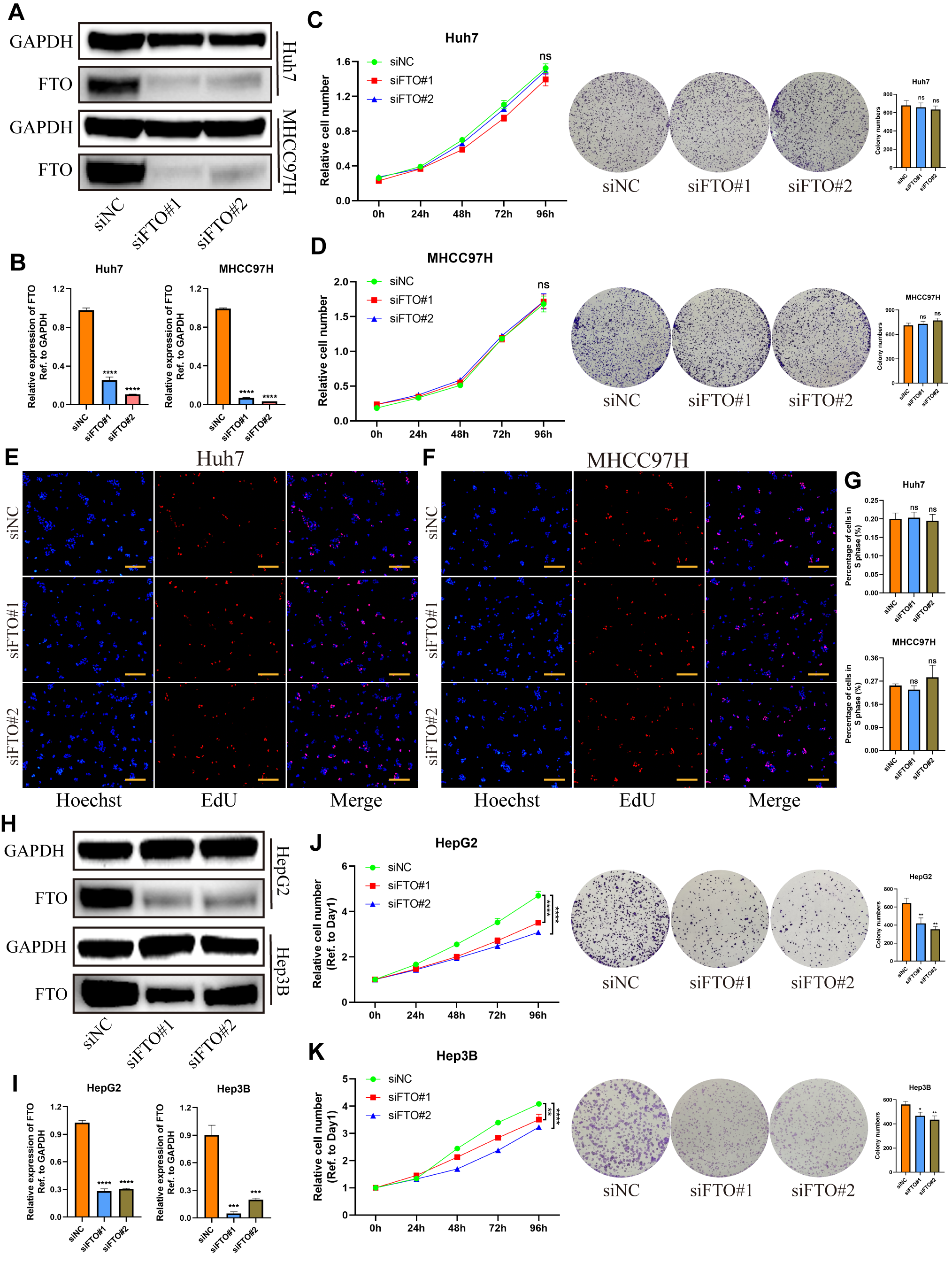

Supplement: Supplementary file 9 — Additional file 9 : Figure S5. Controversial functional roles of FTO in different HCC cells. a and b Knockdown efficiency of FTO in Huh7 and MHCC97H were measured by western blotting (a) and qPCR (b); c and d CCK-8 and colony formation assays were conducted in FTO-silenced Huh7 (c) and MHCC97H (d) cells. Column charts showed colony numbers of each group (right panel). Loss of FTO contributed little to the proliferation abilities of these two cells. e, f and g Negative control and FTO-silenced Huh7 (e) or MHCC97H (f) cells were subject to EdU assays. Percentage of cells in S phase was quantified in column charts (g); h and i Knockdown efficiency of FTO in HepG2 and Hep3B were determined by western blotting (h) and qPCR (i); j and k CCK-8 and colony formation assays were conducted in FTO-knockdown HepG2 (j) and Hep3B (k) cells. Surprisingly, inhibition of FTO suppressed the proliferation capabilities of these two cells. [file 12943_2020_1239_MOESM9_ESM.tif]

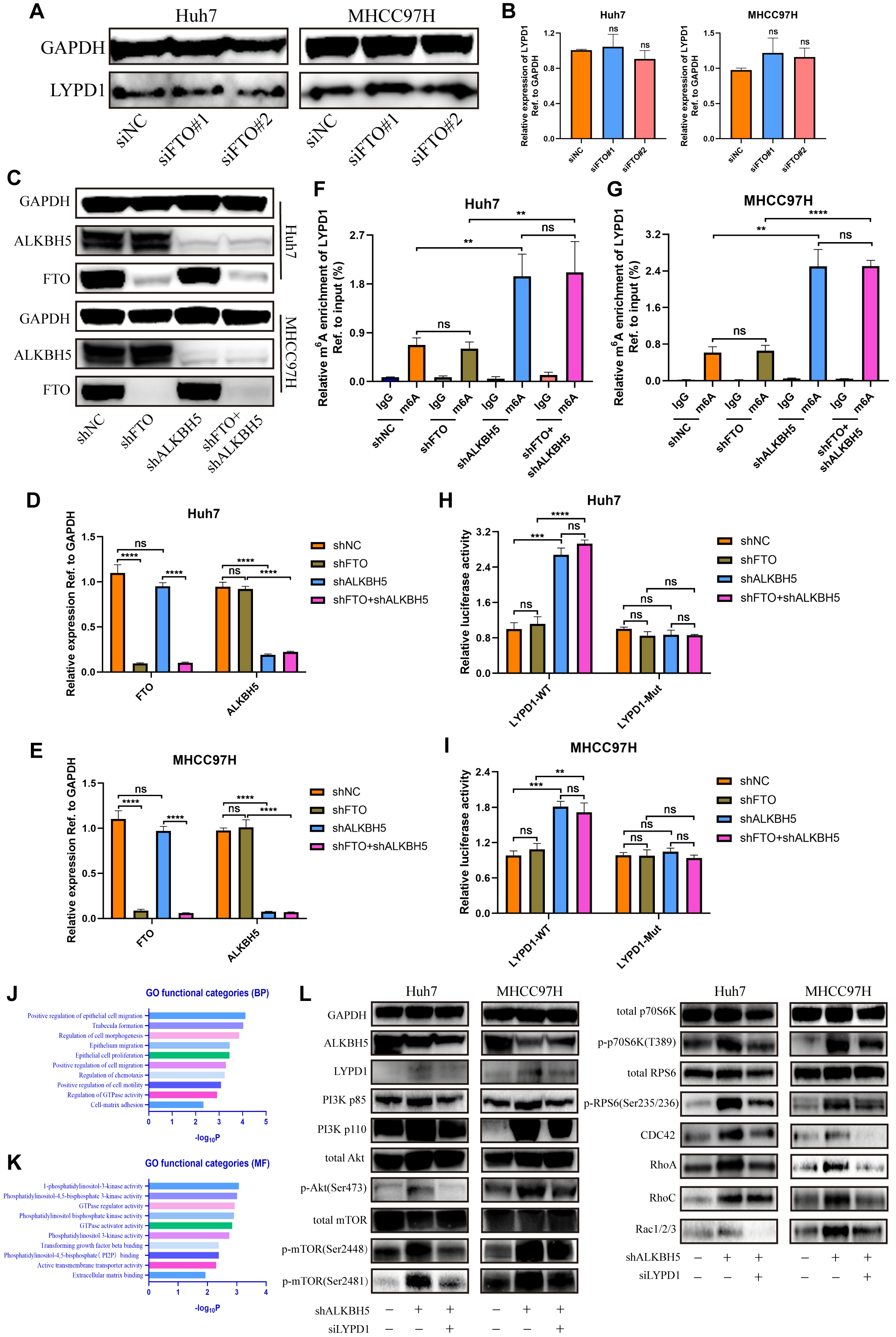

Supplement: Supplementary file 10 — Additional file 10 : Figure S6. The explorations of whether FTO can regulate the m6A modification of LYPD1 and possible downstream pathways of ALKBH5/LYPD1 axis. a and b Expression of LYPD1 was measured when FTO was silenced in Huh7 and MHCC97H cells using western blotting (a) and qPCR (b) assays; c Transfection efficiency was measured by western blotting assays in two HCC cells with individual or double knockdown of FTO and ALKBH5; d and e Transfection efficiency was determined via qPCR assays in Huh7 (d) and MHCC97H (e) cells with individual or double knockdown of FTO and ALKBH5; f and g Relative m6A enrichment of LYPD1 in Huh7 (f) and MHCC97H (g) cells with single or double knockdown of FTO and ALKBH5 were determined by MeRIP-qPCR assays. h and i Relative luciferase activity of Huh7 (h) and MHCC97H (i) cells transfected with the LYPD1-wild type or LYPD1-m6A sites-mutated construct were measured. For each group, FTO and ALKBH5 are individually or double knockdown. j and k GO functional categories containing BP (j) and MF (k) of RNA sequencing using ALKBH5-overexpression or control HCCLM3 cells. When ALKBH5 was overexpressed, those down-regulated transcripts were prominently enriched in BP including epithelial cell migration, cell proliferation or cell adhesion. Meanwhile, they were enriched in MF which mainly contains PI3K activity and GTPase regulator activity; l Western blotting analyses were performed in ALKBH5-silenced HCC cells to check the impacts of ALKBH5 on PI3K-AKT-mTOR and Rho GTPases signaling. Then effects of following LYPD1 inhibition on these pathways were examined. Members of PI3K-AKT-mTOR (including p85, p110, p-AKT, p-mTOR, p-70S6K, p-RPS6) and Rho GTPases (including CDC42, RhoA, RhoC and Rac1/2/3) pathways were tested, respectively. BP: biological processes; MF: molecular function. [file 12943_2020_1239_MOESM10_ESM.tif]
