# Supplementary material for: ALKBH5 suppresses malignancy of hepatocellular carcinoma via m6A-guided epigenetic inhibition of LYPD1
Source: Mol Cancer. 2020 Aug 10;19:123. doi: 10.1186/s12943-020-01239-w (PMC7416417; doi:10.1186/s12943-020-01239-w)

Here, we present the STR certificates for Huh7 (page 2-4), HCCLM3 (page 5-7) and MHCC97H (page 8-17) cell lines, respectively.

中国典型培养物保藏中心
CHINA CENTER FOR TYPE CULTURE COLLECTION (CCTCC)
Wuhan University, Wuhan 430072, China
Phone: 86-027-68752093 **Fax:** 86-027-68754833 **Email:** shenchao@whu.edu.cn

05-21-2019

Entrusted by Zhejiang University, CCTCC has conducted identification experiments on the Huh-7 cell line, and come to the following conclusions:

1. There was no third allele found in Huh-7 cell line, it indicating that there was no cross-contaminant of human source cell line.
2. Compared the STR data of Huh-7 cell line in the databases of ATCC and DSMZ, all the loci of Huh-7 were exactly matched with the loci of HuH-7 (JCRB0403) cells found in DSMZ cell bank, so it is HuH-7 (JCRB0403) cell line (Table 1).

Manager:

China Center for Type Culture Collection

Note:

1. The result is only responsible for the test sample, and the genomic DNA will be reserved for three month.
2. Reference of human cell line authentication: ANSI/ATCC ASN-0002-2011

Table1. The alleles of 21 locations in Huh-7 cell line

Huh-7 cell line（Fig. No. XB19-744）		
Marker	Allele 1	Allele 2
D19S433	13	14
**D5S818**	**12**	**12**
D21S11	30	30
D18S51	15	15
D6S1043	13	15
**AMEL**	**X**	**X**
D3S1358	15	15
**D13S317**	**10**	**11**
**D7S820**	**11**	**11**
**D16S539**	**10**	**10**
**CSF1PO**	**11**	**11**
Penta D	12	12
D2S441	12	14
**vWA**	**16**	**18**
D8S1179	14	14
**TPOX**	**8**	**11**
Penta E	11	11
**TH01**	**7**	**7**
D12S391	20	21
D2S1338	19	19
FGA	22	23

CCTCC

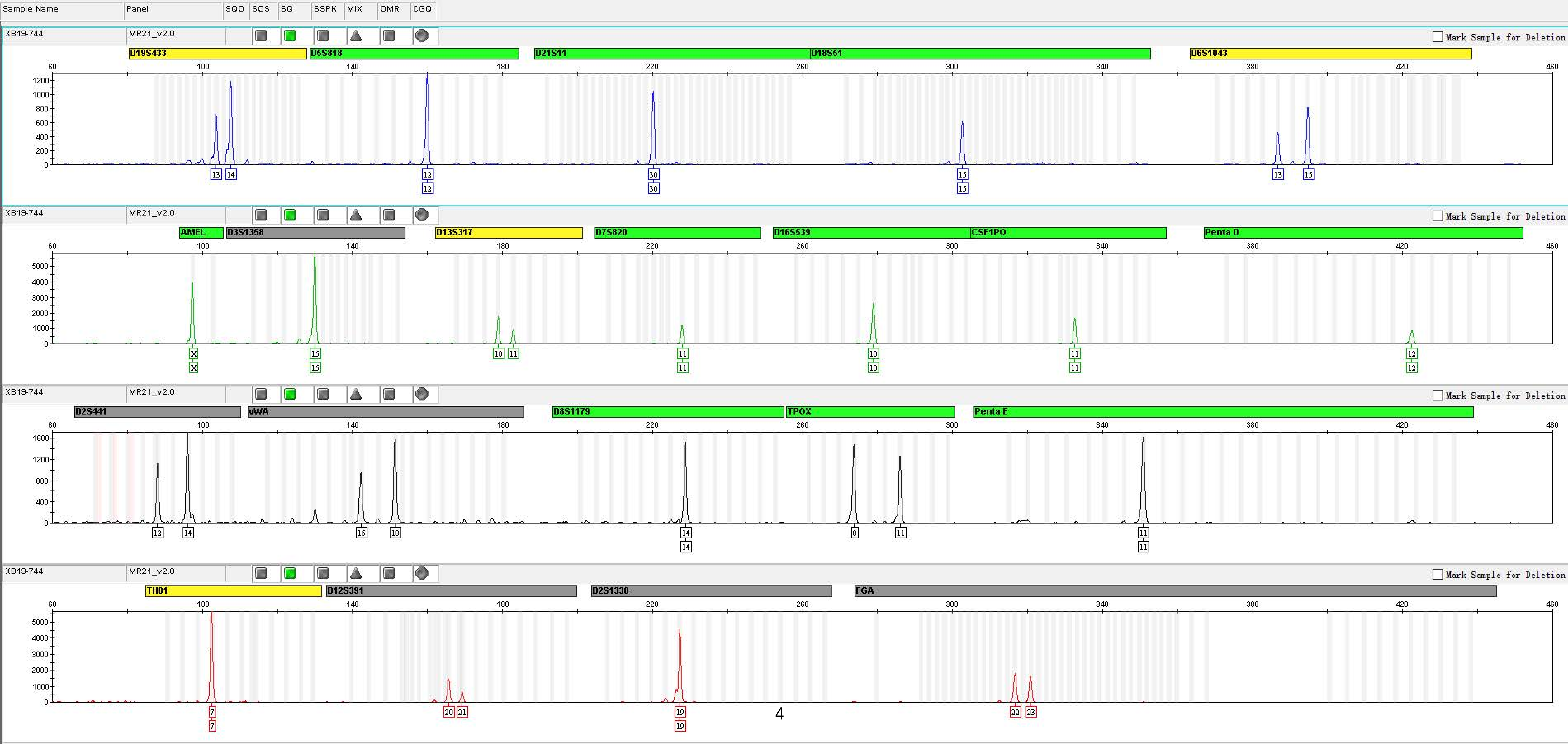
Sample Name
Panel
SQO
SOS
SQ
SSPK
MIX
OMR
CGQ
XB19-744
MR21_v2.0
Mark Sample for Deletion
D19S433
D5S818
D21S11
D18S51
D6S1043
13 14 12 12 30 30 15 15 13 15
AMEL
D3S1358
D13S317
D7S820
D16S539
CSF1PO
Penta D
X X 15 15 10 11 11 11 10 10 11 11 12 12
D2S441
vWA
D8S1179
TPOX
Penta E
12 14 16 18 14 14 8 11 11 11
TH01
D12S391
D2S1338
FGA
7 7 20 21 19 19 22 23

中国典型培养物保藏中心
CHINA CENTER FOR TYPE CULTURE COLLECTION (CCTCC)
Wuhan University, Wuhan 430072, China
Phone: 86-027-68752093 Fax: 86-027-68754833 Email: shenchao@whu.edu.cn

05-23-2019

Entrusted by Zhejiang University, CCTCC has conducted identification experiments on the HCC-LM3 cell line, and come to the following conclusions:

1. There was no third allele found in HCC-LM3 cell line, it indicating that there was no cross-contaminant of human source cell line.
2. Compared the STR data of HCC-LM3 cell line in the databases of ATCC and DSMZ, its profile does not exactly match with any of the current data (Table 1).
3. The STR data of HCC-LM3 cell line and NCI-H82 (HTB-175) cell line matches the highest rate of 73% in ATCC database.

Manager:

China Center for Type Culture Collection

中国典型培养物保藏中心 业务专用章

Note:

1. The result is only responsible for the test sample, and the genomic DNA will be reserved for three month.
2. Reference of human cell line authentication: ANSI/ATCC ASN-0002-2011

Table1. The alleles of 21 locations in HCC-LM3 cell line

HCC-LM3 cell line （Fig. No. XB XB19-750）		
Marker	Allele 1	Allele 2
D19S433	13	14
**D5S818**	**12**	**13**
D21S11	31.2	31.2
D18S51	13	22
D6S1043	12	20
**AMEL**	**X**	**Y**
D3S1358	15	16
**D13S317**	**8**	**8**
**D7S820**	**10**	**10**
**D16S539**	**12**	**12**
**CSF1PO**	**11**	**13**
Penta D	8	9
D2S441	15	15
**vWA**	**14**	**14**
D8S1179	12	13
**TPOX**	**8**	**8**
Penta E	17	17
**TH01**	**9**	**9**
D12S391	18	18
D2S1338	20	20
FGA	21	24

CCTCC

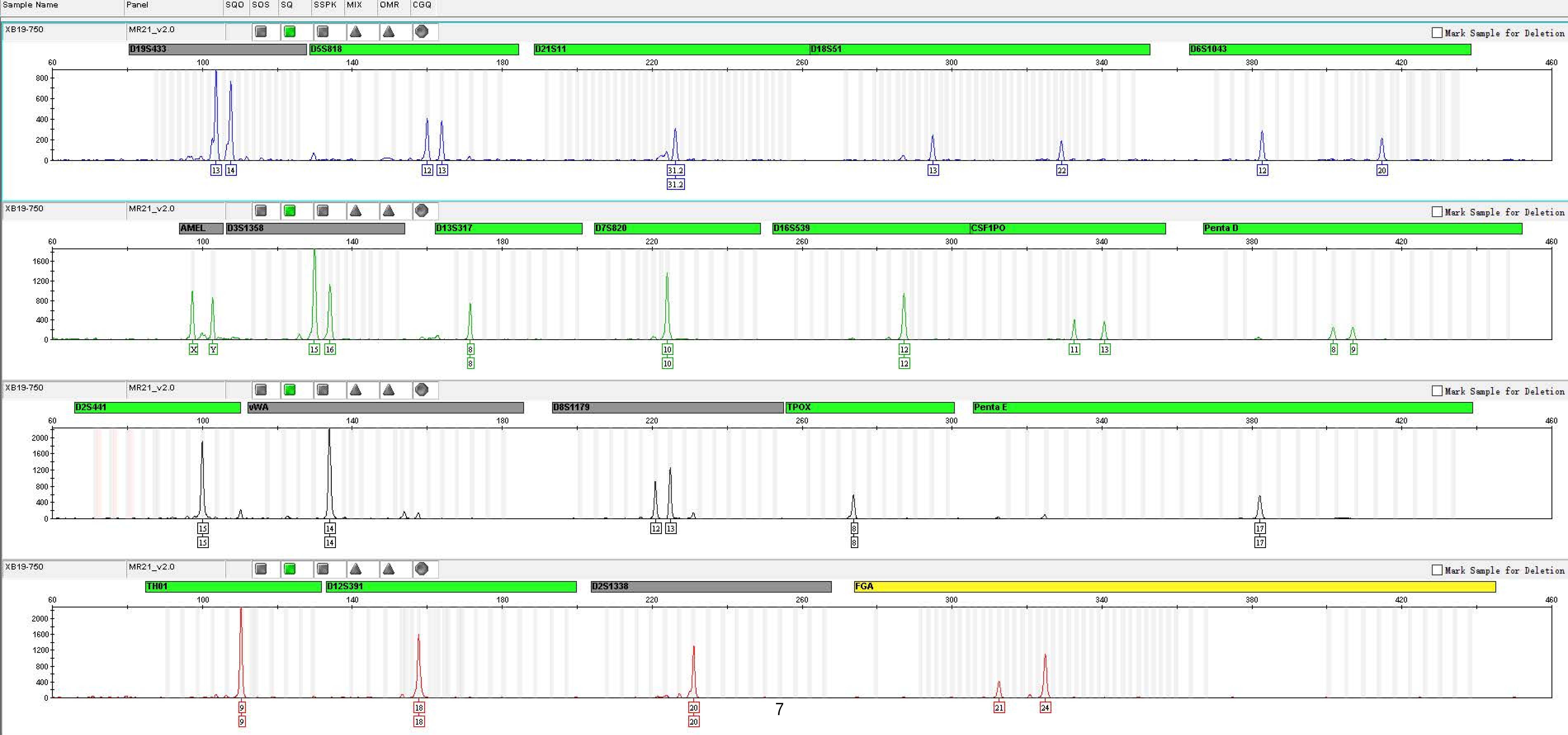
Sample Name
Panel
SQO
SOS
SQ
SSPK
MIX
OMR
CGQ
XB19-750
MR21_v2.0
Mark Sample for Deletion
D19S433
D5S818
D21S11
D18S51
D6S1043
13 14
12 13
31.2
13
22
12 20
AMEL
D3S1358
D13S317
D7S820
D16S539
CSF1PO
Penta D
X Y
15 16
8
10
12
11 13
8 9
D2S441
vWA
D8S1179
TPOX
Penta E
15
14
12 13
8
17
TH01
D12S391
D2S1338
FGA
9
18
20
21 24

细胞遗传质量鉴定检测

Cell Line Authentication Service

STR 基因型检测报告

样品信息

样品编号：

客户样本编号	公司编号
MHCC-97H	20190409-01

样品数量： 1

样品性状： 细胞系

检测项目： STR

检测方法： 用 Axygen 的基因组抽提试剂盒提取 DNA,采用 20- STR 扩增方案扩增,在 ABI 3730XL 型遗传分析仪上对 STR 位点和性别基因 Amelogenin 进行检测。

样品信息

（一）检验基本情况

公司编号	多等位基因	匹配细胞系	细胞库	EV 值	匹配说明
20190409-01	无	-	DSMZ	-	无匹配

样本基因型检验结果

- 多等位基因指三等位及以上基因现象。
- 本次检测各细胞分型结果良好。

（二）各样本描述

- 20190409-01：该株细胞 DNA 分型在细胞系检索中**没有找到匹配**的细胞系，本次检测在该细胞系中没有**发现多等位基因**。（该细胞系未发现多等位基因、未发现交叉污染，细胞系无异常，因数据库未收录 MHCC-97H 相关 STR 信息无法匹配，若为发表论文用，提交该数据给杂志即可）

备注：待测细胞系与收录于 ATCC，DSMZ，JCRB 和 RIKEN 数据库的细胞系 STR 数据进行比对，未收录于以上细胞库的细胞系将无法匹配。

（三）样本分析结果

细胞 20190409-01 的 STR 位点和 Amelogenin 位点的基因分型结果						
Loci	送检细胞 STR 信息			细胞库细胞 STR 信息		
	送检细胞名：MHCC-97H			细胞库细胞名：		
	Allele1	Allele2	Allele3	Allele1	Allele2	Allele3
D5S818	12	13				
D13S317	8	8				
D7S820	10	10				
D16S539	12	12				

VWA	14	14				
TH01	9	9				
AMEL	X	Y				
TPOX	8	8				
CSF1PO	11	13				
D12S391	18	18				
FGA	21	24				
D2S1338	20	20				
D21S11	31.2	31.2				
D18S51	13	22				
D8S1179	12	13				
D3S1358	15	16				
D6S1043	12	20				
PENTAE	11	17				
D19S433	13	14				
PENTAD	8	9				

其他说明

（一）分型方案及位点信息

	方案 1	方案 2	方案 3	方案 4
1	TH01	TPOX	D3S1358	AMEL
2	D12S391	VWA	D13S317	D5S818

3	D7S820	D8S1179	D6S1043	D2S1338
4	CSF1PO	PENTAD	D16S539	D21S11
5	FGA		D19S433	D18S51
6	PENTAE			

实验方案及位点

（二）STR 数据库比对

本公司采用 DSMZ tools 进行细胞系比对,其中包含来自于 ATCC，DSMZ，JCRB 和 RIKEN 数据库的 2455 个细胞系 STR 数据。如果待检测细胞未收录于以上细胞库或这是自行建立的新细胞系将无法进行比对，用户需根据细胞分型结果自行与其他数据库进行比对。

签发日期：2019-04-24

上海传秋生物科技有限公司

Applied Biosystems

GeneMapper 4.0

Cell Line Authentication 1129 1201

Sample File	Sample Name	Panel	OS	SQ
45_E06.fsa	MHCC-97H	STR Profile 1-human		

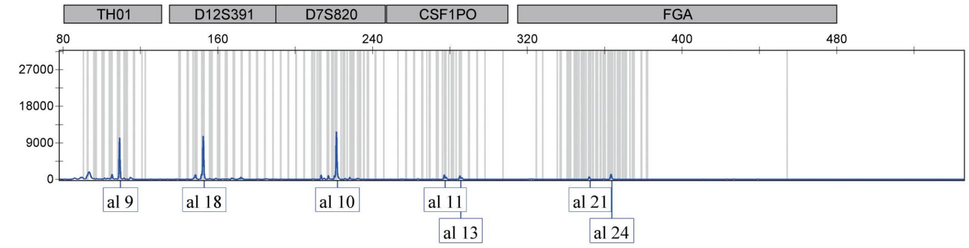

85_E11.fsa	MHCC-97H	STR Profile 2-human		

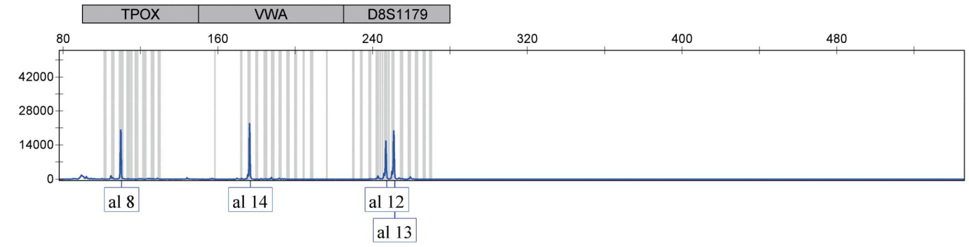

13_E02.fsa	MHCC-97H	STR Profile 3-human		

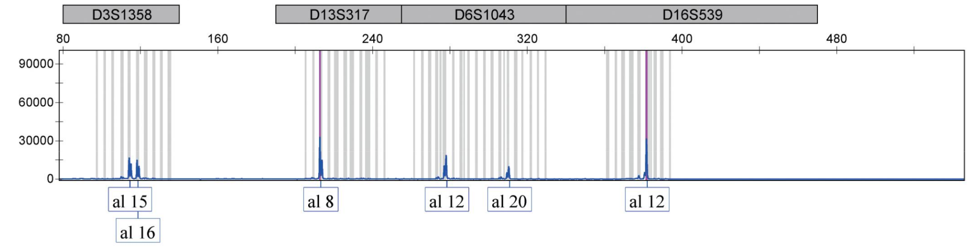

Wed Apr 24,2019 10:15AM, PST | Printed by: gm | Page 1 of 2

Applied Biosystems

GeneMapper 4.0

Cell Line Authentication 1129 1201

Sample File	Sample Name	Panel	OS	SQ
45 E06.fsa	MHCC-97H	STR Profile 4-human		

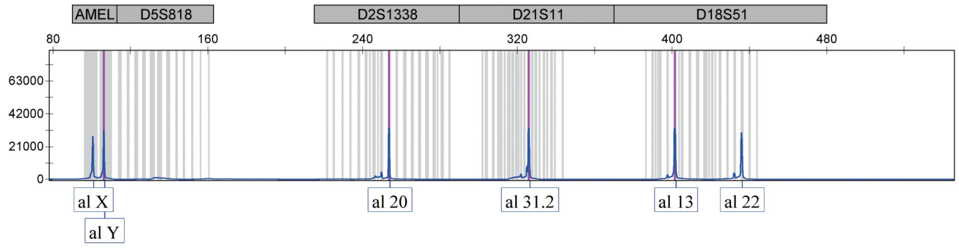

Sample File	Sample Name	Panel	OS	SQ
51 C07 DongJingJing1-5XiBaoJianDing1212.fsa	MHCC-97H	STR Profile 4-human		

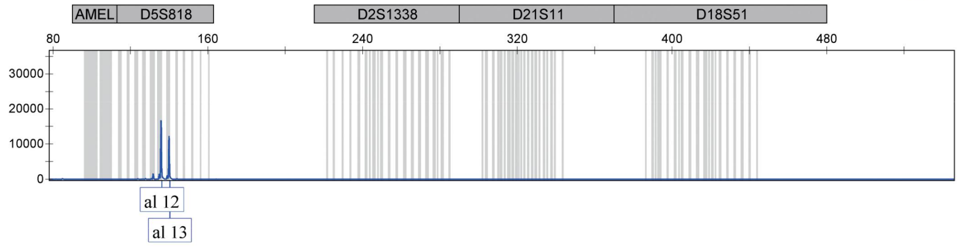

Wed Apr 24,2019 10:15AM, PST   Printed by: gm   Page 2 of 2

www.chuanqiubio.com

Applied Biosystems
GeneMapper 4.0

Cell Line Authentication 1129 1201

Sample File	Sample Name	Panel	OS	SQ
45_E06.fsa	MHCC-97H	STR Profile 1-human		

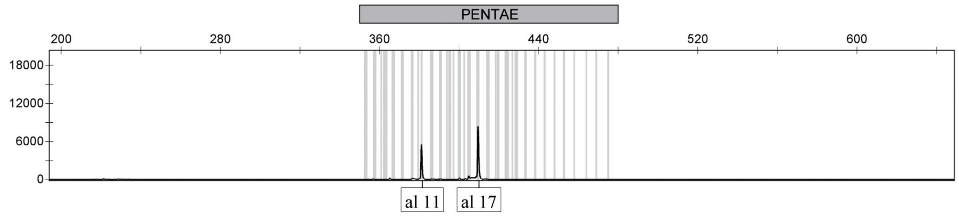

Sample File	Sample Name	Panel	OS	SQ
85_E11.fsa	MHCC-97H	STR Profile 2-human		

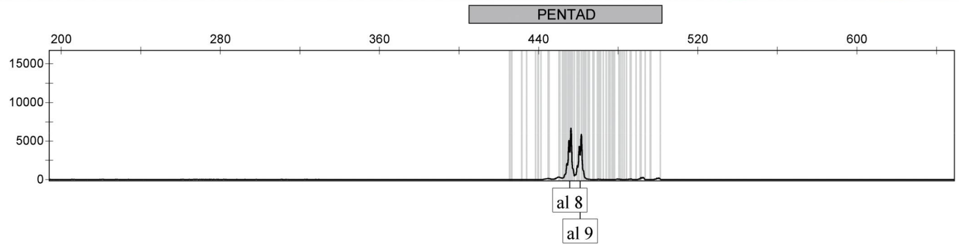

Sample File	Sample Name	Panel	OS	SQ
13_E02.fsa	MHCC-97H	STR Profile 3-human		

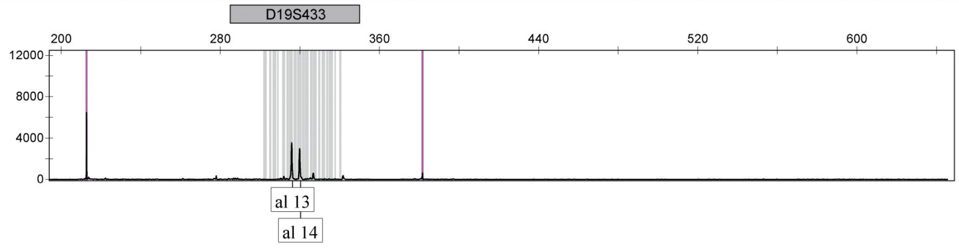

Wed Apr 24,2019 10:16AM, PST　Printed by: gm　Page 1 of 1

上海传秋生物科技有限公司

Applied Biosystems
GeneMapper 4.0

Cell Line Authentication 0908

Sample File	Sample Name	Panel	OS	SQ
03_G03.fsa	yangxing	STR Profile 1-human		

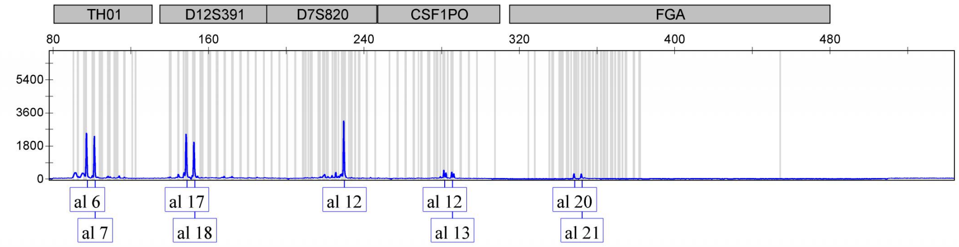

03_G03.fsa	yangxing	STR Profile 1-human		

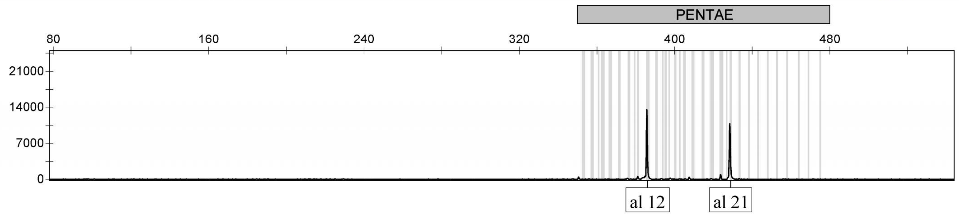

08_H08.fsa	yangxing	STR Profile 2-human		

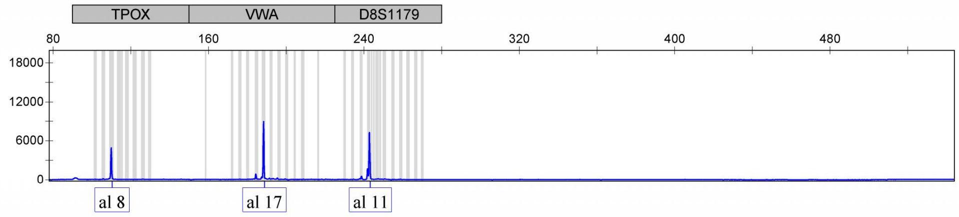

Thu Sep 21,2017 03:18PM, PDT   Printed by: gm   Page 1 of 3

Applied Biosystems

GeneMapper 4.0

Cell Line Authentication 0908

Sample File	Sample Name	Panel	OS	SQ
08_H08.fsa	yangxing	STR Profile 2-human		

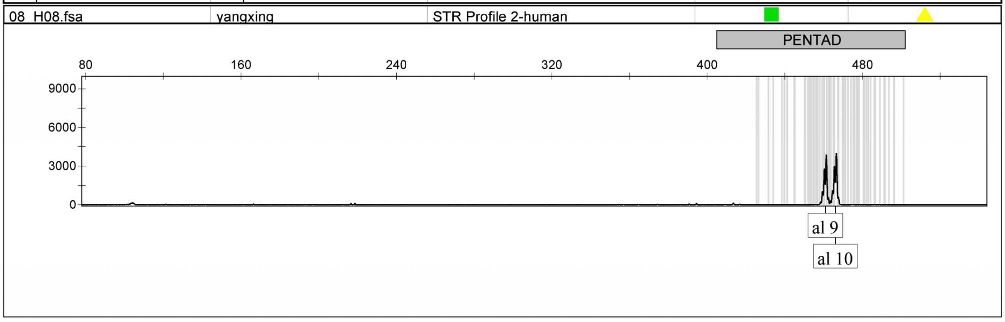

03_F03.fsa	yangxing	STR Profile 3-human		

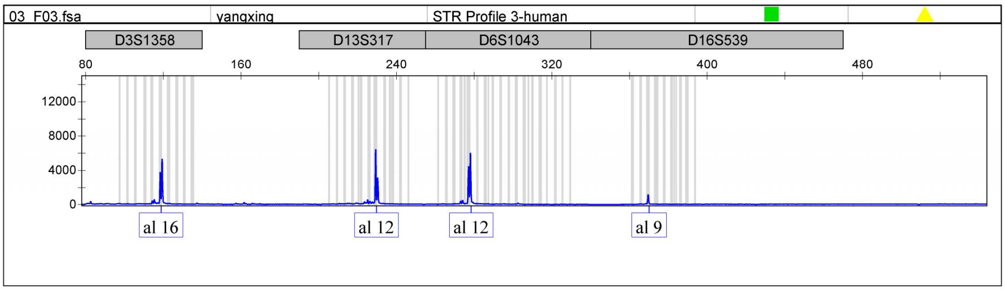

03_F03.fsa	yangxing	STR Profile 3-human		

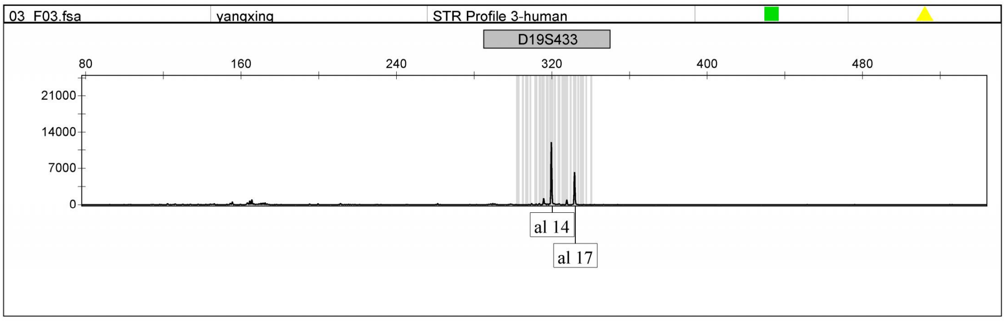

Thu Sep 21,2017 03:18PM, PDT   Printed by: gm   Page 2 of 3

Applied Biosystems

GeneMapper 4.0

Cell Line Authentication 0908

Sample File	Sample Name	Panel	OS	SQ
10_F10.fsa	yangxing	STR Profile 4-human		

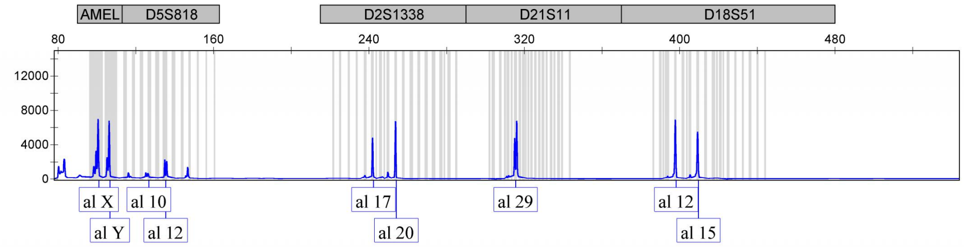

10_F10.fsa	yangxing	STR Profile 4-human		

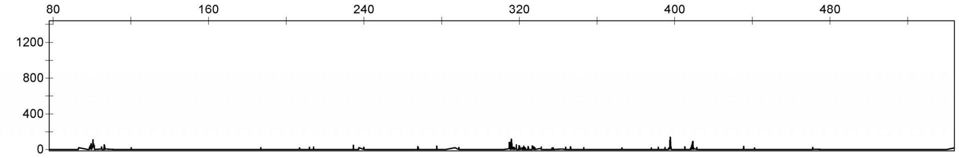

Supplement: Supplementary file 11 — Additional file 11. STR certificates for Huh7 (page 2–4), HCCLM3 (page 5–7) and MHCC97H (page 8–17) cell lines. [file 12943_2020_1239_MOESM11_ESM.pdf]
