# Supplementary material for: ALKBH5 suppresses malignancy of hepatocellular carcinoma via m6A-guided epigenetic inhibition of LYPD1
Source: Mol Cancer. 2020 Aug 10;19:123. doi: 10.1186/s12943-020-01239-w (PMC7416417; doi:10.1186/s12943-020-01239-w)
Supplement: Supplementary file 12 — Additional file 12. Major R codes for MeRIP-seq analyses. [file 12943_2020_1239_MOESM12_ESM.docx]

**Code1. Alignment to human genome using HISAT2**

my $ForkManager = Parallel::ForkManager->new($t_sample);

foreach my $sample(@good2run){

my $pid = $ForkManager->start and next;

my $R1 = $samples{$sample}{1};

my $R2 = $samples{$sample}{2};

my $this_dir = "$tmp_dir/$sample/mapping";

system "mkdir -p $this_dir";

my $argv_rg = "--rg-id $sample --rg SM:$sample --rg LB:$sample --rg PL:illumina --rg PU:$sample";

system "$SOFT_HISAT2 -p $t_soft --dta --dta-cufflink -q -x $DB_HISAT2 -1 $R1 -2 $R2 $argv_rg | $SOFT_SAMTOOLS view -bh | $SOFT_SAMBAMBA s

ort --tmpdir $this_dir -t $t_soft -o $output_dir/$sample.bam /dev/stdin";

$ForkManager->finish;

}

$ForkManager->wait_all_children;

**Code2. Identification of m6A peaks using ExomePeak**

package m6A::different_peak;

use Parallel::ForkManager;

use Excel::Writer::XLSX;

use m6A::format;

use Encode qw/decode/;

sub run

{

my $metadata = shift;

my $base = shift;

my @groups = @{$metadata->{'groups'}};

if (not exists $metadata->{'groups'}) {

print "[Warning] : You must set different peak calling argument in the config.txt!\n";

print "Four columns Separate by blanks!\n";

print "First column : control peak name, sample as peak calling group!\n";

print "Second column : case peak name, sample as peak calling group\n";

print "Third column : control input and ip sample names!\n";

print "Four column : case input and ip sample names\n";

print "Example:\n";

print "Control Case Control-Input;Control-IP Case-Input;Case-IP\n";

exit;

}

my $method = qq{$base->{peak_method}};

my $map = qq{$metadata->{project}/mapping/result};

my $result = qq{$metadata->{project}/diff_peak};

my $peak = qq{$metadata->{project}/peak_calling/result/$method};

my $report = qq{$metadata->{report}/04_Differential_Peak_Analysis};

my $exonpeak = qq{$base->{exonpeak_bin}};

my $chipseeker = qq{$base->{chipseeker_bin}};

my $annovar = qq{$base->{annovar_bin}};

my $meme = qq{$base->{meme_bin}};

my $bedtools = qq{$base->{bedtools_bin}};

my $rscript = qq{$base->{rscript_bin}};

my $sramp = qq{$base->{sramp_bin}};

my $util = qq{$base->{util}};

my $organ = qq{$metadata->{organ}};

my $ref_gtf = qq{$base->{$organ}{genome_mRNA_gtf}};

my $ref_fasta = qq{$base->{$organ}{genome_fasta}};

my $annovar_db = qq{$base->{$organ}{annovar_db}};

my $annovar_version = qq{$base->{$organ}{annovar_version}};

system qq{mkdir -p $result/run} if not -d qq{$result/run};

system qq{mkdir -p $result/result} if not -d qq{$result/result};

system qq{mkdir -p $result/log} if not -d qq{$result/log};

system qq{mkdir -p $report} if not -d $report;

system qq{mkdir -p $report/Genome_Location_Figures} if not -d qq{$report/Genome_Location_Figures};

system qq{mkdir -p $report/Motif_Analysis} if not -d qq{$report/Motif_Analysis};

system qq{mkdir -p $report/Peak_Sequences} if not -d qq{$report/Peak_Sequences};

system qq{mkdir -p $report/Peak_Coordinate_Files} if not -d qq{$report/Peak_Coordinate_Files};

my @res_groups = res_check(qq{$result/result}, \@groups);

if (exists $base->{'force_step'}) {

my @steps = split /,/, $base->{'force_step'};

@res_groups = @groups if 7 ~~ @steps;

}

if (scalar @res_groups == 0) {

print qq{peak 差异分析已经运行完成!\n};

#return 0;

}

pre_check($metadata, $base);

my $max_threads = $base->{'thread_peak'};

my $pm = Parallel::ForkManager->new($max_threads);

foreach my $x (@res_groups) {

my $pid = $pm->start and next;

my $control = $x->[0];

my $case = $x->[1];

my ($control_input, $control_ip) = split /;/, $x->[2];

my ($case_input, $case_ip) = split /;/, $x->[3];

my @control_inputs = split /,/, $control_input;

my @control_ips = split /,/, $control_ip;

my @case_inputs = split /,/, $case_input;

my @case_ips = split /,/, $case_ip;

my $control_input_samples = join ",", map { qq{$map/$_/accepted_hits.bam} } @control_inputs;

my $control_ip_samples = join ",", map { qq{$map/$_/accepted_hits.bam} } @control_ips;

my $case_input_samples = join ",", map { qq{$map/$_/accepted_hits.bam} } @case_inputs;

my $case_ip_samples = join ",", map { qq{$map/$_/accepted_hits.bam} } @case_ips;

my $bams = qq{$control_input_samples,$case_input_samples};

$bams =~ s/,/ /g;

my $profile_samples = qq{$control_input,$case_input};

my $name = qq{$case\_vs_$control};

system qq{mkdir -p $report/Motif_Analysis/$name} if not -d qq{$report/Motif_Analysis/$name};

my $cmd = qq{$exonpeak Rscript $util/exomePeak_diff.R $name $control_input_samples $control_ip_samples $case_input_samples $cas

e_ip_samples $ref_gtf $result/result};

my $filter = qq{perl $util/parse_exomepeak.pl $result/result/$name/diff_peak.xls $result/result/$name/diff_peak.bed $result/resu

lt/$name/filter.diff_peak.xls $result/result/$name/filter.diff_peak.bed};

my $cp_peak_bed = qq{grep -v "#" $result/result/$name/filter.diff_peak.bed | awk '{print \$1"\\t"\$2"\\t"\$3"\\tpeak_"NR"\\t"\$5"

\\t"\$6"\\t"\$7"\\t"\$8"\\t"\$9"\\t"\$10"\\t"\$11"\\t"\$12}' > $report/Peak_Coordinate_Files/$name.diff_peak.bed};

# plot peak on chrom

my $extract_peak_bed = qq{grep -v "#" $result/result/$name/filter.diff_peak.bed >$result/result/$name/for.plot.diff_peak.bed};

my $plot_chr = qq{$chipseeker Rscript $util/peak_on_chr.R $result/result/$name/for.plot.diff_peak.bed $report/Genome_Location_Fi

gures/$name.peak.on.chromsomes.pdf};

# annovar peak bed annotation

my $stat = qq{perl $util/peak_stat.pl -i $result/result/$name/filter.diff_peak.xls -o $result/result/$name/peak.summary -n $name}

;

my $fmt_bed = qq{grep -v "#" $result/result/$name/filter.diff_peak.bed | awk '{print \$1"\\t"\$2"\\t"\$3"\\t0\\t0"}' > $result/re

sult/$name/peak.annovar.bed\n};

my $anno = qq{perl $annovar $result/result/$name/peak.annovar.bed --hgvs --splicing_threshold 0 --neargene 2000 --buildver $anno

var_version $annovar_db\n};

my $merge = qq{perl $util/merge_diff_peak_with_annovar.pl $result/result/$name/filter.diff_peak.xls $result/result/$name/peak.an

novar.bed.variant_function > $result/result/$name/final.xls\n};

my $count = qq{perl $util/chip_region.pl $result/result/$name/final.xls > $result/result/$name/location.count.xls};

my $plot = qq{$rscript Rscript $util/region_pie.R $result/result/$name/location.count.xls $report/Genome_Location_Figures/$name

.genome.region.pdf};

# different peak profie

my $diff_peak_bed = qq{grep -v "Not DE" $result/result/$name/final.xls | awk '{print \$2"\\t"\$3"\\t"\$4"\\t"\$1"\\t"\$8"\\t"\$1

9}' | grep -v "strand" > $result/result/$name/for.profile.bed};

my $sort_bed = qq{sort -k1,1 -k2,2n -k3,3n $result/result/$name/for.profile.bed > $result/result/$name/for.profile.sorted.be

d};

my $count_peak = qq{$bedtools bedtools multicov -bams $bams -bed $result/result/$name/for.profile.sorted.bed > $result/result/

$name/diff.peak.profile.xls};

my $fmt_count = qq{perl $util/peak_profile_fmt.pl $result/result/$name/diff.peak.profile.xls $profile_samples > $result/resul

t/$name/diff.peak.profile.fmt.xls};

# overlap with peak

my $overlap = qq{awk '{print \$2"\\t"\$3"\\t"\$4"\\t"\$1"\\t"\$8}' $result/result/$name/final.xls |grep -v "strand" > $result/res

ult/$name/for.overlap.bed};

my $intersect_control = qq{$bedtools bedtools intersect -a $result/result/$name/for.overlap.bed -b $peak/$control/for.overlap.bed

-wb > $result/result/$name/overlap_with_$control};

my $intersect_case = qq{$bedtools bedtools intersect -a $result/result/$name/for.overlap.bed -b $peak/$case/for.overlap.bed -wb > $resul

t/result/$name/overlap_with_$case};

my $add_overlap = qq{perl $util/add_overlap.pl $result/result/$name/final.xls $result/result/$name/overlap_with_$control $result/result/$

name/overlap_with_$case $control $case > $result/result/$name/result.xls};

my $add_peak_type = qq{perl $util/add_diff_peak_sig.pl $result/result/$name/result.xls $result/result/$name/sig_diff_peak.xls $result/res

ult/$name/con_sig_diff_peak.xls > $result/result/$name/peak.result.xls};

## motif

my $get_fasta = qq{$bedtools bedtools getfasta -s -fi $ref_fasta -bed $result/result/$name/filter.diff_peak.bed -split -fo $r

esult/result/$name/peak.fasta};

my $fmt_fa = qq{perl $util/format_peak_fasta.pl $result/result/$name/final.xls $result/result/$name/peak.fasta > $result/result/$nam

e/peak.fmt.fasta};

my $cp_peak_fasta = qq{cp $result/result/$name/peak.fmt.fasta $report/Peak_Sequences/$name.peak.fasta};

# sramp m6A sites

my $m6A_sites = qq{$sramp $result/result/$name/peak.fmt.fasta $result/result/$name/peak.m6A.sites.xls full"\n};

my $merge_m6A_sites = qq{perl $util/merge_m6A_sites.pl $result/result/$name/peak.m6A.sites.xls $result/result/$name/peak.result.xls > $re

sult/result/$name/peak.final.xls};

my $motif = qq{$meme meme-chip -norc -oc $result/result/$name/meme -meme-p 20 $result/result/$name/peak.fmt.fasta};

my $fmt_fimo = qq{perl $util/format_fimo.pl $result/result/$name/meme > $result/result/$name/fimo.final.xls};

my $cp = qq{cp $result/result/$name/meme/dreme_out/* $report/Motif_Analysis/$name};

my $fmt = qq{perl $util/dreme_out_fmt.pl -i $result/result/$name/meme/dreme_out/dreme.txt -o $result/result/$name/motif.xls};

my $add_motif = qq{perl $util/add_motif.pl $result/result/$name/peak.final.xls $result/result/$name/fimo.final.xls > $result/result/$na

me/peak.result.final.xls};

my $touch = qq{touch $result/result/$name/$name.finish};

open SAVE, qq{>$result/run/$name.sh} or die "Can't open $result/run/$name.sh!\n";

print SAVE qq{$cmd\n};

print SAVE qq{$filter\n$stat\n$fmt_bed\n$anno\n$merge\n$count\n$plot\n};

print SAVE qq{$cp_peak_bed\n};

print SAVE qq{$extract_peak_bed\n$plot_chr\n};

print SAVE qq{$overlap\n$intersect_control\n$intersect_case\n$add_overlap\n};

print SAVE qq{$add_peak_type\n};

print SAVE qq{$get_fasta\n$fmt_fa\n$motif\n$cp\n$fmt\n$fmt_fimo\n};

print SAVE qq{$cp_peak_fasta\n};

print SAVE qq{$m6A_sites\n};

print SAVE qq{$merge_m6A_sites\n};

print SAVE qq{$diff_peak_bed\n$sort_bed\n$count_peak\n$fmt_count\n};

print SAVE qq{$add_motif\n};

print SAVE qq{$touch\n};

close SAVE;

system qq{bash $result/run/$name.sh &> $result/log/$name.log\n};

$pm->finish;

}

$pm->wait_all_children;

print qq{peak 差异分析已经运行完成!\n};

my %hash = ();

my @names = ();

foreach my $x (@groups) {

my $control = $x->[0];

my $case = $x->[1];

my $name = qq{$case\_vs_$control};

push @names, $name;

my $count = qq{$result/result/$name/location.count.xls};

open TXT, $count or die "Can't open $count!\n";

while (<TXT>) {

chomp;

my @arr = split /\t/;

$hash{$arr[0]}{$name} = $arr[1];

}

close TXT;

}

my @type = ("upstream", "exonic", "intronic", "intergenic", "downstream", "UTR5", "UTR3");

open SAVE, qq{>$result/result/genome.count.xls} or die "Can't open $result/result/genome.count.xls!\n";

my $head = join "\t", @names;

print SAVE qq{type\t$head\n};

foreach my $x (@type){

my @res = ($x);

foreach my $y (@names) {

push @res, $hash{$x}{$y};

}

my $Line = join "\t", @res;

print SAVE qq{$Line\n};

}

close SAVE;

system qq{$rscript Rscript $util/stack_barplot.R $result/result/genome.count.xls $report/Genome_Location_Figures/Genome.region.pdf &> /d

ev/null};

my $excel = qq{$report/Differential_Peak_Summary.xlsx};

my $workbook = Excel::Writer::XLSX->new($excel);

my %format = m6A::format::run($workbook);

my $worksheet = $workbook->add_worksheet("summary");

my $title = qq{Group\tTotal\tUp\tDown};

my @head = split /\t/, $title;

$worksheet->write_row( 0, 0, \@head, $format{'title'});

my $row = 1;

foreach my $x (@groups) {

my $control = $x->[0];

my $case = $x->[1];

my $name = qq{$case\_vs_$control};

my $diff_peak = qq{$result/result/$name/final.xls};

my ($total, $up, $down) = cal_num($diff_peak);

my @res = ($name, $total, $up, $down);

$worksheet->write_row( $row, 0, \@res, $format{'normal'});

$row++;

}

foreach my $x (@groups) {

my $control = $x->[0];

my $case = $x->[1];

my $name = qq{$case\_vs_$control};

my $diff_peak = qq{$result/result/$name/peak.result.final.xls};

my $worksheet = $workbook->add_worksheet(qq{$case\_vs_$control});

my $row = 0;

open TXT, $diff_peak or die "Can't open $diff_peak!\n";

while (<TXT>) {

chomp;

my @arr = split /\t/;

if ($row == 0) {

$worksheet->write_row( $row, 0, \@arr, $format{'title'});

} else {

$worksheet->write_row( $row, 0, \@arr, $format{'normal'});

}

$row++;

}

close TXT;

}

my $worksheet1 = $workbook->add_worksheet("README");

my $A1 = decode("utf8", "标题"); my $A2 = decode("utf8", "说明");

my $B1 = decode("utf8", "peak_id"); my $B2 = decode("utf8", "peak编号");

my $C1 = decode("utf8", "chr"); my $C2 = decode("utf8", "RNA甲基化位点所在的染色体名称");

my $D1 = decode("utf8", "chromStart"); my $D2 = decode("utf8", "RNA甲基化位点在染色体上的起始位置");

my $E1 = decode("utf8", "chromEnd"); my $E2 = decode("utf8", "RNA甲基化位点在染色体上的终止位置 ");

my $F1 = decode("utf8", "location"); my $F2 = decode("utf8", "RNA甲基化位点在基因上的区域 ");

my $G1 = decode("utf8", "name"); my $G2 = decode("utf8", "RNA甲基化位点对应的基因名称");

my $H1 = decode("utf8", "motif"); my $H2 = decode("utf8", "peak上存在的motif名称");

my $I1 = decode("utf8", "m6A_sites"); my $I2 = decode("utf8", "软件预测的可能发生甲基化修饰的A碱基的位置");

my $J1 = decode("utf8", "Classification"); my $J2 = decode("utf8", "m6A位点的可能性的分类");

my $K1 = decode("utf8", "overlap_with_control"); my $K2 = decode("utf8", "与control组peak的overlap");

my $L1 = decode("utf8", "overlap_with_case"); my $L2 = decode("utf8", "与case组peak的overlap");

my $M1 = decode("utf8", "peak_type"); my $M2 = decode("utf8", "peak的类别");

my $N1 = decode("utf8", "score"); my $N2 = decode("utf8", "peak 区间对应的p值，越小说明是一个真实的RNA甲基化位点的可能性越大");

my $O1 = decode("utf8", "strand"); my $O2 = decode("utf8", "peak 区间的正负链信息");

my $P1 = decode("utf8", "thickStart"); my $P2 = decode("utf8", "和 ChromStart 的信息完全一样");

my $Q1 = decode("utf8", "thickEnd"); my $Q2 = decode("utf8", "和 ChromEnd 的信息完全一样");

my $R1 = decode("utf8", "itemRdb"); my $R2 = decode("utf8", "总是0，无明确含义");

my $S1 = decode("utf8", "blockCount"); my $S2 = decode("utf8", "peak 区间包含的外显子区间的个数");

my $T1 = decode("utf8", "blockSizes"); my $T2 = decode("utf8", "peak 区间包含的外显子区间的大小，多个外显子区间用逗号分隔");

my $U1 = decode("utf8", "blockStarts"); my $U2 = decode("utf8", "peak 区间包含的外显子区间的起始位置，注意是相对基因的起始位置计算的，多

个外显子用逗号分隔");

my $V1 = decode("utf8", "lg.p"); my $V2 = decode("utf8", "log10 pvalue");

my $W1 = decode("utf8", "lg.fdr"); my $W2 = decode("utf8", "log10 fdr");

my $X1 = decode("utf8", "fold_enrichment"); my $X2 = decode("utf8", "相对INPUT样本的富集倍数");

my $Y1 = decode("utf8", "diff.lg.fdr"); my $Y2 = decode("utf8", "差异检验的FDR值");

my $Z1 = decode("utf8", "diff.lg.p"); my $Z2 = decode("utf8", "差异检验的p值");

my $AA1 = decode("utf8", "diff.log2.fc"); my $AA2 = decode("utf8", "对照组相比实验组的差异倍数");

my $AB1 = decode("utf8", "type"); my $AB2 = decode("utf8", "peak类型，Not DE代表非差异peak, Up代表上调peak, Down代表下调peak");

$worksheet1->write_row( 0, 0, [$A1, $A2], $format{'title'});

$worksheet1->write_row( 1, 0, [$B1, $B2], $format{'normal'});

$worksheet1->write_row( 2, 0, [$C1, $C2], $format{'normal'});

$worksheet1->write_row( 3, 0, [$D1, $D2], $format{'normal'});

$worksheet1->write_row( 4, 0, [$E1, $E2], $format{'normal'});

$worksheet1->write_row( 5, 0, [$F1, $F2], $format{'normal'});

$worksheet1->write_row( 6, 0, [$G1, $G2], $format{'normal'});

$worksheet1->write_row( 7, 0, [$H1, $H2], $format{'normal'});

$worksheet1->write_row( 8, 0, [$I1, $I2], $format{'normal'});

$worksheet1->write_row( 9, 0, [$J1, $J2], $format{'normal'});

$worksheet1->write_row( 10, 0, [$K1, $K2], $format{'normal'});

$worksheet1->write_row( 11, 0, [$L1, $L2], $format{'normal'});

$worksheet1->write_row( 12, 0, [$M1, $M2], $format{'normal'});

$worksheet1->write_row( 13, 0, [$N1, $N2], $format{'normal'});

$worksheet1->write_row( 14, 0, [$O1, $O2], $format{'normal'});

$worksheet1->write_row( 15, 0, [$P1, $P2], $format{'normal'});

$worksheet1->write_row( 16, 0, [$Q1, $Q2], $format{'normal'});

$worksheet1->write_row( 17, 0, [$R1, $R2], $format{'normal'});

$worksheet1->write_row( 18, 0, [$S1, $S2], $format{'normal'});

$worksheet1->write_row( 19, 0, [$T1, $T2], $format{'normal'});

$worksheet1->write_row( 20, 0, [$U1, $U2], $format{'normal'});

$worksheet1->write_row( 21, 0, [$V1, $V2], $format{'normal'});

$worksheet1->write_row( 22, 0, [$W1, $W2], $format{'normal'});

$worksheet1->write_row( 23, 0, [$X1, $X2], $format{'normal'});

$worksheet1->write_row( 24, 0, [$Y1, $Y2], $format{'normal'});

$worksheet1->write_row( 25, 0, [$Z1, $Z2], $format{'normal'});

$worksheet1->write_row( 26, 0, [$AA1, $AA2], $format{'normal'});

$worksheet1->write_row( 27, 0, [$AB1, $AB2], $format{'normal'});

$workbook->close();

my $excel = qq{$report/Motif_Analysis/Motif_Summary.xlsx};

my $workbook = Excel::Writer::XLSX->new($excel);

my %format = m6A::format::run($workbook);

foreach my $x (@groups) {

my $control = $x->[0];

my $case = $x->[1];

my $name = qq{$case\_vs_$control};

my $out_dir = qq{$result/result/$name};

my $worksheet = $workbook->add_worksheet(qq{$case\_vs_$control});

my $row = 0;

open TXT, qq{$out_dir/motif.xls} or die "Can't open $out_dir/motif.xls!\n";

while (<TXT>) {

chomp;

my @arr = split /\t/;

if ($row == 0) {

$worksheet->write_row( $row, 0, \@arr, $format{'title'});

} else {

$worksheet->write_row( $row, 0, \@arr, $format{'normal'});

}

$row++;

}

close TXT;

}

my $worksheet1 = $workbook->add_worksheet("README");

my $A1 = decode("utf8", "标题"); my $A2 = decode("utf8", "说明");

my $B1 = decode("utf8", "Number"); my $B2 = decode("utf8", "motif编号");

my $C1 = decode("utf8", "Motif"); my $C2 = decode("utf8", "motif名字，用序列表示");

my $D1 = decode("utf8", "Word"); my $D2 = decode("utf8", "motif的序列");

my $E1 = decode("utf8", "RC Word"); my $E2 = decode("utf8", "反向互补链上的序列");

my $F1 = decode("utf8", "Pos"); my $F2 = decode("utf8", "正链的次数");

my $G1 = decode("utf8", "Neg"); my $G2 = decode("utf8", "负链的次数");

my $H1 = decode("utf8", "P-value"); my $H2 = decode("utf8", "P值,表征motif的可信度，越小可信度越高");

my $I1 = decode("utf8", "E-value"); my $I2 = decode("utf8", "E值");

$worksheet1->write_row( 0, 0, [$A1, $A2], $format{'title'});

$worksheet1->write_row( 1, 0, [$B1, $B2], $format{'normal'});

$worksheet1->write_row( 2, 0, [$C1, $C2], $format{'normal'});

$worksheet1->write_row( 3, 0, [$D1, $D2], $format{'normal'});

$worksheet1->write_row( 4, 0, [$E1, $E2], $format{'normal'});

$worksheet1->write_row( 5, 0, [$F1, $F2], $format{'normal'});

$worksheet1->write_row( 6, 0, [$G1, $G2], $format{'normal'});

$worksheet1->write_row( 7, 0, [$H1, $H2], $format{'normal'});

$worksheet1->write_row( 8, 0, [$I1, $I2], $format{'normal'});

$workbook->close();

my $excel = qq{$report/Motif_Analysis/Motif_To_Peak.xlsx};

my $workbook = Excel::Writer::XLSX->new($excel);

my %format = m6A::format::run($workbook);

foreach my $x (@groups) {

my $control = $x->[0];

my $case = $x->[1];

my $name = qq{$case\_vs_$control};

my $out_dir = qq{$result/result/$name};

my $worksheet = $workbook->add_worksheet(qq{$case\_vs_$control});

my $row = 0;

open TXT, qq{$out_dir/fimo.final.xls} or die "Can't open $out_dir/fimo.final.xls!\n";

while (<TXT>) {

chomp;

my @arr = split /\t/;

if ($row == 0) {

$worksheet->write_row( $row, 0, \@arr, $format{'title'});

} else {

$worksheet->write_row( $row, 0, \@arr, $format{'normal'});

}

$row++;

}

close TXT;

}

my $worksheet1 = $workbook->add_worksheet("README");

my $A1 = decode("utf8", "标题"); my $A2 = decode("utf8", "说明");

my $B1 = decode("utf8", "motif_id"); my $B2 = decode("utf8", "motifd的名称");

my $C1 = decode("utf8", "motif_alt_id"); my $C2 = decode("utf8", "motif的编号");

my $D1 = decode("utf8", "sequence_name"); my $D2 = decode("utf8", "peak id");

my $E1 = decode("utf8", "start"); my $E2 = decode("utf8", "motif在peak上的起始位置");

my $F1 = decode("utf8", "stop"); my $F2 = decode("utf8", "motif在peak上的终止位置");

my $G1 = decode("utf8", "strand"); my $G2 = decode("utf8", "peak所在基因组的正负链信息");

my $H1 = decode("utf8", "score"); my $H2 = decode("utf8", "软件对motif出现在peak该位置的打分值，数值越大，代表可能性越大");

my $I1 = decode("utf8", "p-value"); my $I2 = decode("utf8", "p值，数值越小，代表结果越具有统计学显著性");

my $J1 = decode("utf8", "q-value"); my $J2 = decode("utf8", "q值，多重假设检验校正之后的数值，数值越小，代表结果越具有统计学显著性");

my $K1 = decode("utf8", "matched_sequence"); my $K2 = decode("utf8", "在peak上出现的符合motfi特征的序列");

$worksheet1->write_row( 0, 0, [$A1, $A2], $format{'title'});

$worksheet1->write_row( 1, 0, [$B1, $B2], $format{'normal'});

$worksheet1->write_row( 2, 0, [$C1, $C2], $format{'normal'});

$worksheet1->write_row( 3, 0, [$D1, $D2], $format{'normal'});

$worksheet1->write_row( 4, 0, [$E1, $E2], $format{'normal'});

$worksheet1->write_row( 5, 0, [$F1, $F2], $format{'normal'});

$worksheet1->write_row( 6, 0, [$G1, $G2], $format{'normal'});

$worksheet1->write_row( 7, 0, [$H1, $H2], $format{'normal'});

$worksheet1->write_row( 8, 0, [$I1, $I2], $format{'normal'});

$worksheet1->write_row( 9, 0, [$J1, $J2], $format{'normal'});

$worksheet1->write_row( 10, 0, [$K1, $K2], $format{'normal'});

$workbook->close();

}

sub pre_check

{

my $metadata = shift;

my $base = shift;

my $util = qq{$base->{util}};

foreach my $x (@groups) {

my $control = $x->[0];

my $case = $x->[1];

my $sample = $x->[2];

my @control_samples = split /,/, (split /;/, $x->[2])[0];

my @case_samples = split /,/, (split /;/, $x->[2])[1];

die qq{$control must have at least one sample!\n} if scalar @control_samples == 0;

die qq{$case must have at least one sample!\n} if scalar @case_samples == 0;

}

}

sub res_check

{

my $out = shift;

my $groups = shift;

my @temp = ();

foreach my $x (@{$groups}) {

my $control = $x->[0];

my $case = $x->[1];

my $name = qq{$case\_vs_$control};

next if -e qq{$out/$name/$name.finish};

push @temp, $x;

}

return @temp;

}

sub cal_num

{

my $xls = shift;

open EOX, $xls or die "Can't open $xls!\n";

my ($total, $up, $down) = (0, 0, 0);

while (<EOX>) {

chomp;

my @arr = split /\t/;

next if $arr[$#arr] eq 'type';

next if $arr[$#arr] =~ /Not DE/;

$total++;

$up++ if $arr[$#arr] eq 'Up';

$down++ if $arr[$#arr] eq 'Down';

}

close EOX;

return ($total, $up, $down);

}

1;

[root@localhost m6A]#

[root@localhost m6A]#

[root@localhost m6A]#

[root@localhost m6A]#

[root@localhost m6A]#

[root@localhost m6A]#

[root@localhost m6A]#

[root@localhost m6A]#

[root@localhost m6A]#

[root@localhost m6A]#

[root@localhost m6A]#

[root@localhost m6A]#

[root@localhost m6A]#

[root@localhost m6A]#

[root@localhost m6A]#

[root@localhost m6A]#

[root@localhost m6A]#

[root@localhost m6A]#

[root@localhost m6A]#

[root@localhost m6A]#

[root@localhost m6A]# more different_peak.pm

package m6A::different_peak;

use Parallel::ForkManager;

use Excel::Writer::XLSX;

use m6A::format;

use Encode qw/decode/;

sub run

{

my $metadata = shift;

my $base = shift;

my @groups = @{$metadata->{'groups'}};

if (not exists $metadata->{'groups'}) {

print "[Warning] : You must set different peak calling argument in the config.txt!\n";

print "Four columns Separate by blanks!\n";

print "First column : control peak name, sample as peak calling group!\n";

print "Second column : case peak name, sample as peak calling group\n";

print "Third column : control input and ip sample names!\n";

print "Four column : case input and ip sample names\n";

print "Example:\n";

print "Control Case Control-Input;Control-IP Case-Input;Case-IP\n";

exit;

}

my $method = qq{$base->{peak_method}};

my $map = qq{$metadata->{project}/mapping/result};

my $result = qq{$metadata->{project}/diff_peak};

my $peak = qq{$metadata->{project}/peak_calling/result/$method};

my $report = qq{$metadata->{report}/04_Differential_Peak_Analysis};

my $exonpeak = qq{$base->{exonpeak_bin}};

my $chipseeker = qq{$base->{chipseeker_bin}};

my $annovar = qq{$base->{annovar_bin}};

my $meme = qq{$base->{meme_bin}};

my $bedtools = qq{$base->{bedtools_bin}};

my $rscript = qq{$base->{rscript_bin}};

my $sramp = qq{$base->{sramp_bin}};

my $util = qq{$base->{util}};

my $organ = qq{$metadata->{organ}};

my $ref_gtf = qq{$base->{$organ}{genome_mRNA_gtf}};

my $ref_fasta = qq{$base->{$organ}{genome_fasta}};

my $annovar_db = qq{$base->{$organ}{annovar_db}};

my $annovar_version = qq{$base->{$organ}{annovar_version}};

system qq{mkdir -p $result/run} if not -d qq{$result/run};

system qq{mkdir -p $result/result} if not -d qq{$result/result};

system qq{mkdir -p $result/log} if not -d qq{$result/log};

system qq{mkdir -p $report} if not -d $report;

system qq{mkdir -p $report/Genome_Location_Figures} if not -d qq{$report/Genome_Location_Figures};

system qq{mkdir -p $report/Motif_Analysis} if not -d qq{$report/Motif_Analysis};

system qq{mkdir -p $report/Peak_Sequences} if not -d qq{$report/Peak_Sequences};

system qq{mkdir -p $report/Peak_Coordinate_Files} if not -d qq{$report/Peak_Coordinate_Files};

my @res_groups = res_check(qq{$result/result}, \@groups);

if (exists $base->{'force_step'}) {

my @steps = split /,/, $base->{'force_step'};

@res_groups = @groups if 7 ~~ @steps;

}

if (scalar @res_groups == 0) {

print qq{peak 差异分析已经运行完成!\n};

#return 0;

}

pre_check($metadata, $base);

my $max_threads = $base->{'thread_peak'};

my $pm = Parallel::ForkManager->new($max_threads);

foreach my $x (@res_groups) {

my $pid = $pm->start and next;

my $control = $x->[0];

my $case = $x->[1];

my ($control_input, $control_ip) = split /;/, $x->[2];

my ($case_input, $case_ip) = split /;/, $x->[3];

my @control_inputs = split /,/, $control_input;

my @control_ips = split /,/, $control_ip;

my @case_inputs = split /,/, $case_input;

my @case_ips = split /,/, $case_ip;

my $control_input_samples = join ",", map { qq{$map/$_/accepted_hits.bam} } @control_inputs;

my $control_ip_samples = join ",", map { qq{$map/$_/accepted_hits.bam} } @control_ips;

my $case_input_samples = join ",", map { qq{$map/$_/accepted_hits.bam} } @case_inputs;

my $case_ip_samples = join ",", map { qq{$map/$_/accepted_hits.bam} } @case_ips;

my $bams = qq{$control_input_samples,$case_input_samples};

$bams =~ s/,/ /g;

my $profile_samples = qq{$control_input,$case_input};

my $name = qq{$case\_vs_$control};

system qq{mkdir -p $report/Motif_Analysis/$name} if not -d qq{$report/Motif_Analysis/$name};

my $cmd = qq{$exonpeak Rscript $util/exomePeak_diff.R $name $control_input_samples $control_ip_samples $case_input_samples $cas

e_ip_samples $ref_gtf $result/result};

my $filter = qq{perl $util/parse_exomepeak.pl $result/result/$name/diff_peak.xls $result/result/$name/diff_peak.bed $result/resu

lt/$name/filter.diff_peak.xls $result/result/$name/filter.diff_peak.bed};

my $cp_peak_bed = qq{grep -v "#" $result/result/$name/filter.diff_peak.bed | awk '{print \$1"\\t"\$2"\\t"\$3"\\tpeak_"NR"\\t"\$5"

\\t"\$6"\\t"\$7"\\t"\$8"\\t"\$9"\\t"\$10"\\t"\$11"\\t"\$12}' > $report/Peak_Coordinate_Files/$name.diff_peak.bed};

# plot peak on chrom

my $extract_peak_bed = qq{grep -v "#" $result/result/$name/filter.diff_peak.bed >$result/result/$name/for.plot.diff_peak.bed};

my $plot_chr = qq{$chipseeker Rscript $util/peak_on_chr.R $result/result/$name/for.plot.diff_peak.bed $report/Genome_Location_Fi

gures/$name.peak.on.chromsomes.pdf};

# annovar peak bed annotation

my $stat = qq{perl $util/peak_stat.pl -i $result/result/$name/filter.diff_peak.xls -o $result/result/$name/peak.summary -n $name}

;

my $fmt_bed = qq{grep -v "#" $result/result/$name/filter.diff_peak.bed | awk '{print \$1"\\t"\$2"\\t"\$3"\\t0\\t0"}' > $result/re

sult/$name/peak.annovar.bed\n};

my $anno = qq{perl $annovar $result/result/$name/peak.annovar.bed --hgvs --splicing_threshold 0 --neargene 2000 --buildver $anno

var_version $annovar_db\n};

my $merge = qq{perl $util/merge_diff_peak_with_annovar.pl $result/result/$name/filter.diff_peak.xls $result/result/$name/peak.an

novar.bed.variant_function > $result/result/$name/final.xls\n};

my $count = qq{perl $util/chip_region.pl $result/result/$name/final.xls > $result/result/$name/location.count.xls};

my $plot = qq{$rscript Rscript $util/region_pie.R $result/result/$name/location.count.xls $report/Genome_Location_Figures/$name

.genome.region.pdf};

# different peak profie

my $diff_peak_bed = qq{grep -v "Not DE" $result/result/$name/final.xls | awk '{print \$2"\\t"\$3"\\t"\$4"\\t"\$1"\\t"\$8"\\t"\$1

9}' | grep -v "strand" > $result/result/$name/for.profile.bed};

my $sort_bed = qq{sort -k1,1 -k2,2n -k3,3n $result/result/$name/for.profile.bed > $result/result/$name/for.profile.sorted.be

d};

my $count_peak = qq{$bedtools bedtools multicov -bams $bams -bed $result/result/$name/for.profile.sorted.bed > $result/result/

$name/diff.peak.profile.xls};

my $fmt_count = qq{perl $util/peak_profile_fmt.pl $result/result/$name/diff.peak.profile.xls $profile_samples > $result/resul

t/$name/diff.peak.profile.fmt.xls};

# overlap with peak

my $overlap = qq{awk '{print \$2"\\t"\$3"\\t"\$4"\\t"\$1"\\t"\$8}' $result/result/$name/final.xls |grep -v "strand" > $result/res

ult/$name/for.overlap.bed};

my $intersect_control = qq{$bedtools bedtools intersect -a $result/result/$name/for.overlap.bed -b $peak/$control/for.overlap.bed

-wb > $result/result/$name/overlap_with_$control};

my $intersect_case = qq{$bedtools bedtools intersect -a $result/result/$name/for.overlap.bed -b $peak/$case/for.overlap.bed -wb > $resul

t/result/$name/overlap_with_$case};

my $add_overlap = qq{perl $util/add_overlap.pl $result/result/$name/final.xls $result/result/$name/overlap_with_$control $result/result/$

name/overlap_with_$case $control $case > $result/result/$name/result.xls};

my $add_peak_type = qq{perl $util/add_diff_peak_sig.pl $result/result/$name/result.xls $result/result/$name/sig_diff_peak.xls $result/res

ult/$name/con_sig_diff_peak.xls > $result/result/$name/peak.result.xls};

## motif

my $get_fasta = qq{$bedtools bedtools getfasta -s -fi $ref_fasta -bed $result/result/$name/filter.diff_peak.bed -split -fo $r

esult/result/$name/peak.fasta};

my $fmt_fa = qq{perl $util/format_peak_fasta.pl $result/result/$name/final.xls $result/result/$name/peak.fasta > $result/result/$nam

e/peak.fmt.fasta};

my $cp_peak_fasta = qq{cp $result/result/$name/peak.fmt.fasta $report/Peak_Sequences/$name.peak.fasta};

# sramp m6A sites

my $m6A_sites = qq{$sramp $result/result/$name/peak.fmt.fasta $result/result/$name/peak.m6A.sites.xls full"\n};

my $merge_m6A_sites = qq{perl $util/merge_m6A_sites.pl $result/result/$name/peak.m6A.sites.xls $result/result/$name/peak.result.xls > $re

sult/result/$name/peak.final.xls};

my $motif = qq{$meme meme-chip -norc -oc $result/result/$name/meme -meme-p 20 $result/result/$name/peak.fmt.fasta};

my $fmt_fimo = qq{perl $util/format_fimo.pl $result/result/$name/meme > $result/result/$name/fimo.final.xls};

my $cp = qq{cp $result/result/$name/meme/dreme_out/* $report/Motif_Analysis/$name};

my $fmt = qq{perl $util/dreme_out_fmt.pl -i $result/result/$name/meme/dreme_out/dreme.txt -o $result/result/$name/motif.xls};

my $add_motif = qq{perl $util/add_motif.pl $result/result/$name/peak.final.xls $result/result/$name/fimo.final.xls > $result/result/$na

me/peak.result.final.xls};

my $touch = qq{touch $result/result/$name/$name.finish};

open SAVE, qq{>$result/run/$name.sh} or die "Can't open $result/run/$name.sh!\n";

print SAVE qq{$cmd\n};

print SAVE qq{$filter\n$stat\n$fmt_bed\n$anno\n$merge\n$count\n$plot\n};

print SAVE qq{$cp_peak_bed\n};

print SAVE qq{$extract_peak_bed\n$plot_chr\n};

print SAVE qq{$overlap\n$intersect_control\n$intersect_case\n$add_overlap\n};

print SAVE qq{$add_peak_type\n};

print SAVE qq{$get_fasta\n$fmt_fa\n$motif\n$cp\n$fmt\n$fmt_fimo\n};

print SAVE qq{$cp_peak_fasta\n};

print SAVE qq{$m6A_sites\n};

print SAVE qq{$merge_m6A_sites\n};

print SAVE qq{$diff_peak_bed\n$sort_bed\n$count_peak\n$fmt_count\n};

print SAVE qq{$add_motif\n};

print SAVE qq{$touch\n};

close SAVE;

system qq{bash $result/run/$name.sh &> $result/log/$name.log\n};

$pm->finish;

}

$pm->wait_all_children;

print qq{peak 差异分析已经运行完成!\n};

my %hash = ();

my @names = ();

foreach my $x (@groups) {

my $control = $x->[0];

my $case = $x->[1];

my $name = qq{$case\_vs_$control};

push @names, $name;

my $count = qq{$result/result/$name/location.count.xls};

open TXT, $count or die "Can't open $count!\n";

while (<TXT>) {

chomp;

my @arr = split /\t/;

$hash{$arr[0]}{$name} = $arr[1];

}

close TXT;

}

my @type = ("upstream", "exonic", "intronic", "intergenic", "downstream", "UTR5", "UTR3");

open SAVE, qq{>$result/result/genome.count.xls} or die "Can't open $result/result/genome.count.xls!\n";

my $head = join "\t", @names;

print SAVE qq{type\t$head\n};

foreach my $x (@type){

my @res = ($x);

foreach my $y (@names) {

push @res, $hash{$x}{$y};

}

my $Line = join "\t", @res;

print SAVE qq{$Line\n};

}

close SAVE;

system qq{$rscript Rscript $util/stack_barplot.R $result/result/genome.count.xls $report/Genome_Location_Figures/Genome.region.pdf &> /d

ev/null};

my $excel = qq{$report/Differential_Peak_Summary.xlsx};

my $workbook = Excel::Writer::XLSX->new($excel);

my %format = m6A::format::run($workbook);

my $worksheet = $workbook->add_worksheet("summary");

my $title = qq{Group\tTotal\tUp\tDown};

my @head = split /\t/, $title;

$worksheet->write_row( 0, 0, \@head, $format{'title'});

my $row = 1;

foreach my $x (@groups) {

my $control = $x->[0];

my $case = $x->[1];

my $name = qq{$case\_vs_$control};

my $diff_peak = qq{$result/result/$name/final.xls};

my ($total, $up, $down) = cal_num($diff_peak);

my @res = ($name, $total, $up, $down);

$worksheet->write_row( $row, 0, \@res, $format{'normal'});

$row++;

}

foreach my $x (@groups) {

my $control = $x->[0];

my $case = $x->[1];

my $name = qq{$case\_vs_$control};

my $diff_peak = qq{$result/result/$name/peak.result.final.xls};

my $worksheet = $workbook->add_worksheet(qq{$case\_vs_$control});

my $row = 0;

open TXT, $diff_peak or die "Can't open $diff_peak!\n";

while (<TXT>) {

chomp;

my @arr = split /\t/;

if ($row == 0) {

$worksheet->write_row( $row, 0, \@arr, $format{'title'});

} else {

$worksheet->write_row( $row, 0, \@arr, $format{'normal'});

}

$row++;

}

close TXT;

}

my $worksheet1 = $workbook->add_worksheet("README");

my $A1 = decode("utf8", "标题"); my $A2 = decode("utf8", "说明");

my $B1 = decode("utf8", "peak_id"); my $B2 = decode("utf8", "peak编号");

my $C1 = decode("utf8", "chr"); my $C2 = decode("utf8", "RNA甲基化位点所在的染色体名称");

my $D1 = decode("utf8", "chromStart"); my $D2 = decode("utf8", "RNA甲基化位点在染色体上的起始位置");

my $E1 = decode("utf8", "chromEnd"); my $E2 = decode("utf8", "RNA甲基化位点在染色体上的终止位置 ");

my $F1 = decode("utf8", "location"); my $F2 = decode("utf8", "RNA甲基化位点在基因上的区域 ");

my $G1 = decode("utf8", "name"); my $G2 = decode("utf8", "RNA甲基化位点对应的基因名称");

my $H1 = decode("utf8", "motif"); my $H2 = decode("utf8", "peak上存在的motif名称");

my $I1 = decode("utf8", "m6A_sites"); my $I2 = decode("utf8", "软件预测的可能发生甲基化修饰的A碱基的位置");

my $J1 = decode("utf8", "Classification"); my $J2 = decode("utf8", "m6A位点的可能性的分类");

my $K1 = decode("utf8", "overlap_with_control"); my $K2 = decode("utf8", "与control组peak的overlap");

my $L1 = decode("utf8", "overlap_with_case"); my $L2 = decode("utf8", "与case组peak的overlap");

my $M1 = decode("utf8", "peak_type"); my $M2 = decode("utf8", "peak的类别");

my $N1 = decode("utf8", "score"); my $N2 = decode("utf8", "peak 区间对应的p值，越小说明是一个真实的RNA甲基化位点的可能性越大");

my $O1 = decode("utf8", "strand"); my $O2 = decode("utf8", "peak 区间的正负链信息");

my $P1 = decode("utf8", "thickStart"); my $P2 = decode("utf8", "和 ChromStart 的信息完全一样");

my $Q1 = decode("utf8", "thickEnd"); my $Q2 = decode("utf8", "和 ChromEnd 的信息完全一样");

my $R1 = decode("utf8", "itemRdb"); my $R2 = decode("utf8", "总是0，无明确含义");

my $S1 = decode("utf8", "blockCount"); my $S2 = decode("utf8", "peak 区间包含的外显子区间的个数");

my $T1 = decode("utf8", "blockSizes"); my $T2 = decode("utf8", "peak 区间包含的外显子区间的大小，多个外显子区间用逗号分隔");

my $U1 = decode("utf8", "blockStarts"); my $U2 = decode("utf8", "peak 区间包含的外显子区间的起始位置，注意是相对基因的起始位置计算的，多

个外显子用逗号分隔");

my $V1 = decode("utf8", "lg.p"); my $V2 = decode("utf8", "log10 pvalue");

my $W1 = decode("utf8", "lg.fdr"); my $W2 = decode("utf8", "log10 fdr");

my $X1 = decode("utf8", "fold_enrichment"); my $X2 = decode("utf8", "相对INPUT样本的富集倍数");

my $Y1 = decode("utf8", "diff.lg.fdr"); my $Y2 = decode("utf8", "差异检验的FDR值");

my $Z1 = decode("utf8", "diff.lg.p"); my $Z2 = decode("utf8", "差异检验的p值");

my $AA1 = decode("utf8", "diff.log2.fc"); my $AA2 = decode("utf8", "对照组相比实验组的差异倍数");

my $AB1 = decode("utf8", "type"); my $AB2 = decode("utf8", "peak类型，Not DE代表非差异peak, Up代表上调peak, Down代表下调peak");

$worksheet1->write_row( 0, 0, [$A1, $A2], $format{'title'});

$worksheet1->write_row( 1, 0, [$B1, $B2], $format{'normal'});

$worksheet1->write_row( 2, 0, [$C1, $C2], $format{'normal'});

$worksheet1->write_row( 3, 0, [$D1, $D2], $format{'normal'});

$worksheet1->write_row( 4, 0, [$E1, $E2], $format{'normal'});

$worksheet1->write_row( 5, 0, [$F1, $F2], $format{'normal'});

$worksheet1->write_row( 6, 0, [$G1, $G2], $format{'normal'});

$worksheet1->write_row( 7, 0, [$H1, $H2], $format{'normal'});

$worksheet1->write_row( 8, 0, [$I1, $I2], $format{'normal'});

$worksheet1->write_row( 9, 0, [$J1, $J2], $format{'normal'});

$worksheet1->write_row( 10, 0, [$K1, $K2], $format{'normal'});

$worksheet1->write_row( 11, 0, [$L1, $L2], $format{'normal'});

$worksheet1->write_row( 12, 0, [$M1, $M2], $format{'normal'});

$worksheet1->write_row( 13, 0, [$N1, $N2], $format{'normal'});

$worksheet1->write_row( 14, 0, [$O1, $O2], $format{'normal'});

$worksheet1->write_row( 15, 0, [$P1, $P2], $format{'normal'});

$worksheet1->write_row( 16, 0, [$Q1, $Q2], $format{'normal'});

$worksheet1->write_row( 17, 0, [$R1, $R2], $format{'normal'});

$worksheet1->write_row( 18, 0, [$S1, $S2], $format{'normal'});

$worksheet1->write_row( 19, 0, [$T1, $T2], $format{'normal'});

$worksheet1->write_row( 20, 0, [$U1, $U2], $format{'normal'});

$worksheet1->write_row( 21, 0, [$V1, $V2], $format{'normal'});

$worksheet1->write_row( 22, 0, [$W1, $W2], $format{'normal'});

$worksheet1->write_row( 23, 0, [$X1, $X2], $format{'normal'});

$worksheet1->write_row( 24, 0, [$Y1, $Y2], $format{'normal'});

$worksheet1->write_row( 25, 0, [$Z1, $Z2], $format{'normal'});

$worksheet1->write_row( 26, 0, [$AA1, $AA2], $format{'normal'});

$worksheet1->write_row( 27, 0, [$AB1, $AB2], $format{'normal'});

$workbook->close();

my $excel = qq{$report/Motif_Analysis/Motif_Summary.xlsx};

my $workbook = Excel::Writer::XLSX->new($excel);

my %format = m6A::format::run($workbook);

foreach my $x (@groups) {

my $control = $x->[0];

my $case = $x->[1];

my $name = qq{$case\_vs_$control};

my $out_dir = qq{$result/result/$name};

my $worksheet = $workbook->add_worksheet(qq{$case\_vs_$control});

my $row = 0;

open TXT, qq{$out_dir/motif.xls} or die "Can't open $out_dir/motif.xls!\n";

while (<TXT>) {

chomp;

my @arr = split /\t/;

if ($row == 0) {

$worksheet->write_row( $row, 0, \@arr, $format{'title'});

} else {

$worksheet->write_row( $row, 0, \@arr, $format{'normal'});

}

$row++;

}

close TXT;

}

my $worksheet1 = $workbook->add_worksheet("README");

my $A1 = decode("utf8", "标题"); my $A2 = decode("utf8", "说明");

my $B1 = decode("utf8", "Number"); my $B2 = decode("utf8", "motif编号");

my $C1 = decode("utf8", "Motif"); my $C2 = decode("utf8", "motif名字，用序列表示");

my $D1 = decode("utf8", "Word"); my $D2 = decode("utf8", "motif的序列");

my $E1 = decode("utf8", "RC Word"); my $E2 = decode("utf8", "反向互补链上的序列");

my $F1 = decode("utf8", "Pos"); my $F2 = decode("utf8", "正链的次数");

my $G1 = decode("utf8", "Neg"); my $G2 = decode("utf8", "负链的次数");

my $H1 = decode("utf8", "P-value"); my $H2 = decode("utf8", "P值,表征motif的可信度，越小可信度越高");

my $I1 = decode("utf8", "E-value"); my $I2 = decode("utf8", "E值");

$worksheet1->write_row( 0, 0, [$A1, $A2], $format{'title'});

$worksheet1->write_row( 1, 0, [$B1, $B2], $format{'normal'});

$worksheet1->write_row( 2, 0, [$C1, $C2], $format{'normal'});

$worksheet1->write_row( 3, 0, [$D1, $D2], $format{'normal'});

$worksheet1->write_row( 4, 0, [$E1, $E2], $format{'normal'});

$worksheet1->write_row( 5, 0, [$F1, $F2], $format{'normal'});

$worksheet1->write_row( 6, 0, [$G1, $G2], $format{'normal'});

$worksheet1->write_row( 7, 0, [$H1, $H2], $format{'normal'});

$worksheet1->write_row( 8, 0, [$I1, $I2], $format{'normal'});

$workbook->close();

my $excel = qq{$report/Motif_Analysis/Motif_To_Peak.xlsx};

my $workbook = Excel::Writer::XLSX->new($excel);

my %format = m6A::format::run($workbook);

foreach my $x (@groups) {

my $control = $x->[0];

my $case = $x->[1];

my $name = qq{$case\_vs_$control};

my $out_dir = qq{$result/result/$name};

my $worksheet = $workbook->add_worksheet(qq{$case\_vs_$control});

my $row = 0;

open TXT, qq{$out_dir/fimo.final.xls} or die "Can't open $out_dir/fimo.final.xls!\n";

while (<TXT>) {

chomp;

my @arr = split /\t/;

if ($row == 0) {

$worksheet->write_row( $row, 0, \@arr, $format{'title'});

} else {

$worksheet->write_row( $row, 0, \@arr, $format{'normal'});

}

$row++;

}

close TXT;

}

my $worksheet1 = $workbook->add_worksheet("README");

my $A1 = decode("utf8", "标题"); my $A2 = decode("utf8", "说明");

my $B1 = decode("utf8", "motif_id"); my $B2 = decode("utf8", "motifd的名称");

my $C1 = decode("utf8", "motif_alt_id"); my $C2 = decode("utf8", "motif的编号");

my $D1 = decode("utf8", "sequence_name"); my $D2 = decode("utf8", "peak id");

my $E1 = decode("utf8", "start"); my $E2 = decode("utf8", "motif在peak上的起始位置");

my $F1 = decode("utf8", "stop"); my $F2 = decode("utf8", "motif在peak上的终止位置");

my $G1 = decode("utf8", "strand"); my $G2 = decode("utf8", "peak所在基因组的正负链信息");

my $H1 = decode("utf8", "score"); my $H2 = decode("utf8", "软件对motif出现在peak该位置的打分值，数值越大，代表可能性越大");

my $I1 = decode("utf8", "p-value"); my $I2 = decode("utf8", "p值，数值越小，代表结果越具有统计学显著性");

my $J1 = decode("utf8", "q-value"); my $J2 = decode("utf8", "q值，多重假设检验校正之后的数值，数值越小，代表结果越具有统计学显著性");

my $K1 = decode("utf8", "matched_sequence"); my $K2 = decode("utf8", "在peak上出现的符合motfi特征的序列");

$worksheet1->write_row( 0, 0, [$A1, $A2], $format{'title'});

$worksheet1->write_row( 1, 0, [$B1, $B2], $format{'normal'});

$worksheet1->write_row( 2, 0, [$C1, $C2], $format{'normal'});

$worksheet1->write_row( 3, 0, [$D1, $D2], $format{'normal'});

$worksheet1->write_row( 4, 0, [$E1, $E2], $format{'normal'});

$worksheet1->write_row( 5, 0, [$F1, $F2], $format{'normal'});

$worksheet1->write_row( 6, 0, [$G1, $G2], $format{'normal'});

$worksheet1->write_row( 7, 0, [$H1, $H2], $format{'normal'});

$worksheet1->write_row( 8, 0, [$I1, $I2], $format{'normal'});

$worksheet1->write_row( 9, 0, [$J1, $J2], $format{'normal'});

$worksheet1->write_row( 10, 0, [$K1, $K2], $format{'normal'});

$workbook->close();

}

sub pre_check

{

my $metadata = shift;

my $base = shift;

my $util = qq{$base->{util}};

foreach my $x (@groups) {

my $control = $x->[0];

my $case = $x->[1];

my $sample = $x->[2];

my @control_samples = split /,/, (split /;/, $x->[2])[0];

my @case_samples = split /,/, (split /;/, $x->[2])[1];

die qq{$control must have at least one sample!\n} if scalar @control_samples == 0;

die qq{$case must have at least one sample!\n} if scalar @case_samples == 0;

}

}

sub res_check

{

my $out = shift;

my $groups = shift;

my @temp = ();

foreach my $x (@{$groups}) {

my $control = $x->[0];

my $case = $x->[1];

my $name = qq{$case\_vs_$control};

next if -e qq{$out/$name/$name.finish};

push @temp, $x;

}

return @temp;

}

sub cal_num

{

my $xls = shift;

open EOX, $xls or die "Can't open $xls!\n";

my ($total, $up, $down) = (0, 0, 0);

while (<EOX>) {

chomp;

my @arr = split /\t/;

next if $arr[$#arr] eq 'type';

next if $arr[$#arr] =~ /Not DE/;

$total++;

$up++ if $arr[$#arr] eq 'Up';

$down++ if $arr[$#arr] eq 'Down';

}

close EOX;

return ($total, $up, $down);

}

1;

**Code3. GO and KEGG analyses**

docker run --rm -v /:$soft_docker_root_dir $soft_docker Rscript $soft_cluster_profiler -s $Species $soft_docker_root_dir/$gene_list $soft

_docker_root_dir/$output";

**Code4. Differential expression analysis**

dgelist <- DGEList(counts = countData, group = condition)

dgelist_norm <- calcNormFactors(dgelist, method = 'TMM')

design <- model.matrix(~condition)

";

if($sample_num == 2){

my %hash;

open FILE, $ref_gene;

while(my $line = <FILE>){

$line =~ s/[\r\n]//g;

my @split = split /\t/, $line;

$hash{$split[1]} = "$split[1]\n$split[12]\n";

}

close FILE;

open SAVE, ">$output_dir/house_keeping_genes.txt";

open FILE, $house_keeping_genes;

while(my $line = <FILE>){

$line =~ s/[\r\n]//g;

my @split = split /\t/, $line;

next if(not exists $hash{$split[3]});

print SAVE $hash{$split[3]};

}

close FILE;

close SAVE;

$r_script .= "

# 读取管家基因

house_keeping_genes <- read.table('$output_dir/house_keeping_genes.txt', header=F, sep='\\t', comment.char='', check.names=F)

# 管家基因计算离散值

y <- dgelist_norm

y\$samples\$group <- 1

y <- estimateDisp(y[which(rownames(y) %in% house_keeping_genes\$V1),], trend = 'none', tagwise = FALSE)

dgelist_norm\$common.dispersion <- y\$common.dispersion

# 广义线性模型

fit <- glmFit(dgelist_norm, design, robust = TRUE)

test <- glmLRT(fit)

";

}else{

$r_script .= "

# 计算离散值

dge <- estimateDisp(dgelist_norm, design, robust = TRUE)

# 精确检验

test <- exactTest(dge)

";

}

$r_script .= "

# 保存结果

write.table(cpm(dgelist_norm), '$output_dir/norm_expression_profile.txt', sep='\\t', quote=F, col.names = NA)

write.table(topTags(test, n=nrow(test)), '$output_dir/test.txt', sep='\\t', quote=F, col.names = NA)

";

open SAVE, ">$output_dir/edgeR.r"; print SAVE $r_script; close SAVE;

system "$SOFT_R_SCRIPT $output_dir/edgeR.r";

my %norm = read_file("$output_dir/norm_expression_profile.txt");

my %test = read_file("$output_dir/test.txt");

my @case_samples = split /,/, $case_samples;

my @control_samples = split /,/, $control_samples;

open SAVE, ">$output_dir/differential_expression.txt";

print SAVE (join "\t", "", @case_samples, @control_samples, "baseMeanA", "baseMeanB", "baseMean", "log2FoldChange", "pvalue", "type", "lo

gCPM", "FDR")."\n";

open FILE, $profile;

<FILE>;

while(my $line = <FILE>){

my $gene = (split /\t/, $line, 2)[0];

next if(not exists $norm{$gene});

my ($sumA, $countA, $sumB, $countB) = (0, 0, 0, 0);

print SAVE $gene;

foreach my $sample(@case_samples){

my $norm = $norm{$gene}{$sample};

print SAVE "\t$norm";

$sumA += $norm;

$countA++;

}

foreach my $sample(@control_samples){

my $norm = $norm{$gene}{$sample};

print SAVE "\t$norm";

$sumB += $norm;

$countB++;

}

my $log2FoldChange = -$test{$gene}{"logFC"};

my $pvalue = $test{$gene}{"PValue"};

my $type = "Not DE";

$type = "Up" if($pvalue < 0.05 and $log2FoldChange >= $log2fc);

$type = "Down" if($pvalue < 0.05 and $log2FoldChange <= -($log2fc));

print SAVE "\t".(join "\t", $sumA/$countA, $sumB/$countB, ($sumA+$sumB)/($countA+$countB), $log2FoldChange, $pvalue, $type, $test

{$gene}{"logCPM"}, $test{$gene}{"FDR"})."\n";

}

close SAVE;

close FILE;

}
